# Combining theory and usability testing to inform optimization and implementation of an online primary care depression management tool

**DOI:** 10.1186/s12911-024-02733-7

**Published:** 2025-01-15

**Authors:** Nicola McCleary, Justin Presseau, Isabelle Perkins, Brittany Mutsaers, Claire E. Kendall, Janet Yamada, Katharine Gillis, Douglas Green

**Affiliations:** 1 Centre for Implementation Research, Clinical Epidemiology Program, Ottawa Hospital Research Institute, Ottawa, Canada; 2 School of Epidemiology and Public Health, University of Ottawa, Ottawa, Canada; 3 Child Health Evaluative Sciences Program, The Hospital for Sick Children - Research Institute, 686 Bay St, Toronto, ON M5G 0A4 Canada; 4 Institute of Health Policy, Management and Evaluation, University of Toronto, Toronto, Canada; 5 School of Psychology, University of Ottawa, Ottawa, Canada; 6 C.T. Lamont Primary Health Care Research Centre, Bruyère Research Institute, Ottawa, Canada; 7 Department of Family Medicine, University of Ottawa, Ottawa, Canada; 8 Clinical Epidemiology Program, Ottawa Hospital Research Institute, Ottawa, Canada; 9 Daphne Cockwell School of Nursing, Toronto Metropolitan University, Toronto, Canada; 10 Department of Psychiatry, University of Ottawa, Ottawa, Canada; 11 Department of Psychiatry, The Ottawa Hospital, Ottawa, Canada

**Keywords:** Depression, Primary care professionals, Behaviour change, Usability, Qualitative methods, Implementation

## Abstract

**Background:**

The ‘Ottawa Depression Algorithm’ is an evidence-based online tool developed to support primary care professionals care for adults with depression. Uptake of such tools require provider behaviour change. Identifying issues which may impact use of an innovation in routine practice (i.e. barriers to and enablers of behaviour change) informs the selection of implementation strategies that can be deployed with the tool to support use. However, established theory-informed barriers/enablers assessment methods may be less well suited to identifying issues with tool usability. User testing methods can help to determine whether the tool itself is optimally designed. We aimed to integrate these two methodological approaches to i) identify issues impacting the usability of algorithm; and ii) identify barriers to and enablers of algorithm use in routine practice.

**Methods:**

We conducted semi-structured interviews with primary care professionals in Ottawa, Canada. To evaluate usability, participants used a written patient scenario to work through the algorithm while verbalizing their thoughts (‘Think Aloud’). Participants were then asked about factors influencing algorithm use in routine practice (informed by the Theoretical Domains Framework). We used the codebook approach to thematic analysis to assign statements to pre-specified codes and develop themes pertaining to usability and routine use.

**Results:**

We interviewed 20 professionals from seven practices. Usability issues were summarised within five themes: *Optimizing content and flow to align with issues faced in practice*, *Enhancing the most useful algorithm components*, *Interactivity of the algorithm and embedded tools*, *Clarity of presence, purpose, or function of components*, and *Navigational challenges and functionality of links*. Barriers to and enablers of routine use were summarised within five themes: *Getting to know the algorithm*, *Alignment with roles and pathways of influence*, *Integration with current ways of working*, *Contexts for use*, and *Anticipated benefits and concerns about patient communication*.

**Conclusions:**

Whilst the Ottawa Depression Algorithm was viewed as a useful tool, specific usability issues and barriers to use were identified. Supplementing a theory-based barriers/enablers assessment with usability testing provided enhanced insights to inform optimization and implementation of this clinical tool. We have provided a methods guide for others who may wish to apply this approach.

**Supplementary Information:**

The online version contains supplementary material available at 10.1186/s12911-024-02733-7.

## Background

Depression is the second leading cause of global disability [1]. Primary care settings are often the first point of contact for those experiencing symptoms of depression [2–4]. However, challenges to care provision include time constraints, lack of experience or expertise in supporting patients with different levels of depressive severity, and lack of mental health care resources, which can lead to suboptimal care [5–7]. The “Ottawa Depression Algorithm” [8], an online tool grounded in clinical guidelines and evidence-based practices [9], was developed to support primary care professionals in providing care for adult patients presenting with symptoms of depression. The algorithm presents an interactive clinical pathway to help with assessment, diagnosis, and treatment according to severity, and contains links to resources and appropriate treatment avenues.

Uptake of the algorithm in routine practice will require primary care professionals to change their typical practice behaviour, in order to integrate algorithm use into their existing workflows and care processes. Implementation science focuses on understanding the best ways to move evidence into practice [10], a key component of which is understanding factors that impede change (barriers) or support change (enablers). Using evidence-supported behaviour change theories to guide barriers and enablers assessments allows us to draw on what is already known about factors which influence behaviour [11]. Barriers and enablers assessments are often informed by the Theoretical Domains Framework (TDF) [12, 13]. Developed from a synthesis of theories, the TDF comprises 14 factors (‘domains’) that influence healthcare professional behaviour. The TDF focuses on both internal (e.g. knowledge, skills, motivation, self-efficacy) and external (e.g. organizational, physical and social) influences and can be used to identify key barriers and enablers which can then be addressed by specific strategies to move evidence into practice via behaviour change [14, 15].

Whilst theory-informed barriers and enablers assessments can identify issues which may impact uptake of an innovation into routine practice, they may be less well-suited to identifying specific issues which impact the usability of a new clinical tool. User-centred design provides a framework for developing products (including new clinical tools) which starts with understanding the end-users of the product and the needs that the product is intended to fulfil [16, 17]. It includes a set of methods for developing products whereby end-users are involved to influence aspects of the design to optimize their interaction with the product and ultimately improve product effectiveness [16, 18]. The set of methods comprise different ways to involve users in the design process [18]. Usability testing is one such method, which “involves hands-on evaluation of the extent to which a product or innovation can be used by specified users to achieve specified goals” [19]. This can help to identify usability issues and as such, usability testing is another approach gaining recognition for the value it can add within the field of implementation science.

Combining these two methodological approaches may be useful in situations where supporting healthcare professional behaviour change to move evidence into practice includes the integration of a new clinical tool. In this study, we present an illustrative example of combining usability testing with a theory-informed barriers and enablers assessment. Our aims were to i) identify issues impacting the usability of the Ottawa Depression Algorithm by primary care professionals; and ii) identify barriers to and enablers of the use the Ottawa Depression Algorithm in routine primary care to support the diagnosis and treatment of adult patients presenting with symptoms of depression.

## Methods

### Study design

We conducted a qualitative study comprising semi-structured, one-to-one, in person interviews.

### Setting

This study took place in primary care practices in the Champlain Local Health Integration Network (now called Home and Community Care Support Services Champlain) in the Province of Ontario, Canada. In Ontario, primary care is publicly funded: permanent residents are insured for medically necessary hospital and physician services through the Ontario Health Insurance Plan, and primary care visits are free at the point of care [20].

### Online tool development

The algorithm was designed to be used in primary care to treat and support adult patients with depression at the point of care. It was developed by psychiatrists based at The Ottawa Hospital and the University of Ottawa. Development began by mapping the typical pathway by which patients are diagnosed and treated in primary care through consultation with family physicians. Next, a psychiatry resident integrated recommendations from evidence-based clinical guidelines [1, 9]. Further input was provided by a colleague who developed an online tool to support access to mental health help (www.ementalhealth.ca). Resources were embedded into the pathway to support providers (e.g. diagnostic tools, a process for selecting medication, information about where to refer patients for psychotherapy and other community supports). Primary care colleagues were consulted throughout the development process. The algorithm was initially designed as a PDF document and then transformed into an online tool with the aim of improving accessibility and reach.

### Participants and recruitment

Eligible participants were family physicians, residents in Family Medicine specialty training, nurses, nurse practitioners, and administrators working in primary care settings. We purposively recruited participants to ensure a mix of individuals with differing familiarity with the tool and differing levels of experience in primary care. DG (a psychiatrist and developer of the algorithm) emailed existing contacts at local practices with a request to distribute information about the study. NMc emailed individuals who indicated interest to invite them to participate and arrange a time for the interview. Up to two reminders emails were sent. Participants were informed that completion of the interview was taken as implied informed consent to participate. Informed verbal consent was also obtained at the beginning of each interview. As a token of appreciation, participants were offered entry into a prize draw to win one of two $200 gift cards.

We aimed to recruit participants until we achieved data saturation for the theory-based barriers and enablers assessment. We applied the ‘10 + 3 rule’ whereby at least 10 interviews were conducted and analyzed, followed by additional sets of three interviews: when the additional three interviews did not raise any new shared beliefs, we would take this as evidence of saturation [21].

### Data collection

An interview guide was developed to facilitate interview processes (Additional File 1). The interview comprised two parts. Part one focused on usability testing. Participants were asked to work through the algorithm while “thinking aloud”. The Think-Aloud method involves participants verbalizing their thoughts while completing a task (in this case, using the algorithm to provide care for a patient with symptoms of depression described in a written scenario) [22, 23]. Initial patient scenarios were drafted by BM and then refined by NMc and checked for clinical realism by DG and CK. The final scenario used (Additional File 2) was presented to participants in two parts, representing an initial consultation and a follow-up visit. Part two of the interview focused on exploring barriers to and enablers of using the algorithm in routine practice. Interview questions were informed by the TDF and associated guidance for its application [12, 13, 24]. The TDF was selected because it is theory-based, focuses on factors which are modifiable and can therefore be addressed with interventions, and because it was developed specifically to support understanding of healthcare provider behaviour. The interview process was piloted with primary care providers and subsequently optimized. Interviews were conducted by NMc in-person and audio-recorded. As the Ottawa Depression Algorithm is available as an online resource, access to a computer and an internet connection were required to access the algorithm during the interview.

### Data analysis

Digital audio files were transcribed verbatim by a third party. Transcripts were de-identified and assigned a unique study number. NMc reviewed the accuracy of the transcriptions before proceeding with the analyses. This step also facilitated familiarisation with the data. Transcripts were imported into NVivo (QSR International) qualitative analysis software and analyzed using the codebook approach to thematic analysis [25, 26]. One researcher (NMc) coded the transcripts using a codebook (Additional File 3) which listed codes representing key usability categories [27–31] and the TDF domains [12, 13]. The usability categories and their descriptions were drawn from two sources: descriptions of established methodological approaches for usability testing of medical informatics innovations such as clinical decision support tools (categories such as workflow, content, usefulness, understandability, visibility, and navigation) [27–29], and evidence-based guidelines developed to support the design of information-oriented websites (categories such as layout or organization, links, search, graphics, and hardware or software) [30, 31]. These sources were chosen since the algorithm is a clinical decision support tool delivered in a website format. Meaningful units of text within transcripts were assigned to one or more of the usability categories/TDF domains. Coding was discussed with JP and DG as this initial analysis progressed, and refined accordingly. A second researcher (IP) then reviewed and coded the transcripts containing the first researcher’s coding. The two researchers met regularly to discuss areas of disagreement, reach consensus, and update the codebook where appropriate. One researcher (NMc) then reviewed the text coded within each usability category and TDF domain and developed belief statements to represent responses with a similar underlying belief that suggested an influence on the target behaviour (use of the algorithm) [24]. One researcher (NMc) then developed themes across usability categories and TDF domains by reviewing the belief statements and considering how they may be combined to form over-arching patterns of shared meaning which are coherent around a central concept [26]. Themes were refined through discussion with another researcher (JP).

## Results

### Participants

We interviewed 20 participants from seven practices. Participant characteristics are summarised in Table 1. Half were family physicians, and half were nurse practitioners, residents, or administrators. No new shared beliefs concerning barriers to or enablers of algorithm use were identified in interviews 13, 14, and 15, meeting our definition of data saturation: however, we conducted five more interviews to increase the variation in participant roles. All participants practiced in interdisciplinary team-based models of care. Twelve were not aware of the algorithm, six were aware but hadn’t used it, and two had some experience using it.

**Table 1:** Participant characteristics (*n* = 20)

Characteristic		Number (%) of participants
Role	Family physician	10 (50)
	Nurse practitioner	7 (35)
	Resident	2 (10)
	Administrator	1 (5)
Practice type	Academic Family Health Team	7 (35)
	Non-Academic Family Health Team	8 (40)
	Community Health Centre	5 (25)
Years qualified (mean (standard deviation), range)		13.9 (12.3), 1–41

### Usability testing

Participants viewed the algorithm and its content positively, thought it was user-friendly, and were enthusiastic about its potential to support them in providing depression care. For example, participants said *“I like the quality of the resources”* (p19); *“I love that when you open things up, it gives you things” (p06)*; *“It’s very comprehensive and user-friendly, you just click button and all the information will show up”* (p07); “*To actually be able to go through this* [medication side effects content] *myself or with the patient would be really helpful… otherwise it’s more of an abstract conversation so this is really nice for a lot of my patients. It’s really good actually.”* (p15).

**Fig. 1:**
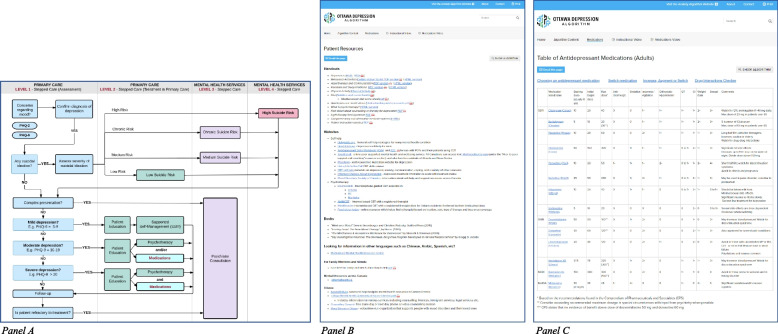
Ottawa Depression Algorithm – example content. Panel **A** The interactive clinical pathway at the core of the algorithm and presented on the homepage. Clicking the boxes with solid borders takes users to further information on the stated topic, as presented in Panels B and C. This version was viewed by study participants and has since been updated. Panel **B** The patient resources section (formerly labelled the patient education section). This is the current version which was not viewed by participants but is similar in content and structure to the version they viewed. Panel **C** The medications section. This is the current version which was not viewed by participants but is similar in content and structure to the version they viewed

However, specific issues impacting usability were identified and coded within the following usability categories: Workflow, Content, Usefulness, Understandability, Completeness, Layout or organisation, Visibility, Navigation, and Links. Key issues were grouped into five themes, described below. Table 2 presents sample quotes for each theme. Usability categories included in the codebook but not represented in these themes are listed in Additional File 4 with a rationale for exclusion. Figure 1 provides example algorithm content to help situate the usability findings.

**Table 2:** Relevant categories, key usability issues, and sample quotes for five over-arching themes regarding algorithm usability

Category	Key usability issues (*n*)	Sample quotes
***Theme 1: Optimizing content and flow to align with issued faced in practice***		
Content	Mention of specific content that aligns with practice (11), for example: - ‘Patient education’ box (4) - ‘Confirm diagnosis of depression’ box (3) - Individual medication algorithms (3)	“Patient education we’re clicking on the green box. We’re looking at the handouts section. Just running down the list of stuff that’s available. Okay so the book Mind Over Mood is one that I have, and I refer patients to, so that’s good. Websites we’ve used Mood Gym, we’ve used Living Life to the Full as well.” (p08)
	Mention of specific content that doesn ’t align with practice (6), for example: - ‘Patient education’ box (3)	“Maybe it’s my own character, but I can’t imagine my patients using this in a way to change their behaviour. Reading something doesn’t enable people to change, they have to practice something, and so workshops are much more effective than a handout” (p18)
Completeness	More treatment options needed for mild depression (3)	“I’m a bit surprised there’s no medication box for the mild depression because they might not need antidepressant per se, but they might need something to sleep, and if we don’t treat the insomnia sometimes it makes them more anxious or more depressed… so I think I would probably add another medication box here for mild, not necessarily for the mild depression but for the other symptoms” (p14)
	No info or resources for geriatric depression (3)	“when I have a geriatric patient, I tend to use the Geriatric Depression Score… I didn’t see it.” (p16)
Workflow	‘Email this page’ function: some misalignment with current patient communication practices (3)	“It [the function] is helpful in principle. We don’t use email to communicate with patients here. We use something called the patient portal. And the reason is if you type in the wrong email address you can send sensitive messages to the wrong person… But I wonder if something like that could be part of the portal. We can certainly send attachments through the portal. So, as long as this were available as a list of attachments, we could just click the ones we need.” (p02)
Layout or organisation	Complex presentation box link to psychiatry consultation—not always done in practice (7)	“I feel that I don’t always initiate a referral to psychiatry. But I think it’s probably good to have it in there, if yes is meant to be ‘yes consider’? If yes is meant to be ‘you must’, then I’m not sure I agree with that. I’m not sure sending a person with a personality disorder who’s depressed to psychiatry straightaway, without seeing how they respond to the therapeutic intervention makes any sense… we work a lot with learners and some of them are very concrete, where they see a ‘yes’, that means you must. And so it might be interesting to put like ‘yes, consider’ or ‘if so, consider’” (p09)
Workflow	Placement of anxiety does not align with clinical workflow (2)	“I usually do a PHQ9 and a GAD7 at the same time when I’m assessing depression… I would have not put anxiety here, I might have wanted to put that up as part of the initial assessment… And put the regular PHQ9 and GAD7 as an element.” (p18)
***Theme 2: Enhancing the most useful algorithm components***		
Content	Mention of specific content which is good (17), for example: - Medications Table (7) - Patient education (6) - ‘Email this page’ function (5)	“it also has the options for medications. I’m just gonna look at this. The starting dose, initial target, max, this is good, and then some of the major side effects. And then the main things to watch for, it’s really good.” (p04)
Links	‘Medications ’ page—SwitchRx link is good (4)	“Oh switch medications, oh yeah I’m familiar with SwitchRx, that’s helpful I’m glad it’s there.” (p18)
Usefulness	Mention of specific tools or components which appear helpful or useful (17), for example: - Patient resources (11) - Medications Sect. (9)	“Medications, so this is helpful in terms of starting doses… Choosing an antidepressant medication. Has the patient had a particular response to a certain antidepressant in the past? Has there been a family history or response to a certain… Oh so this is actually very helpful in terms of choosing which one… So I know the basics of this, but this is actually really helpful and it ‘s outlined really well” (p16)
Completeness	Medications table—no info on avg time to impact (2)	“Another thing that I might like to see in here, and would be helpful to show them (patients), would be the average time for it to take to work. I know just from using them that some are a lot faster than others, and everyone’s different, but for example, when they might be able to expect to see some sort of improvement.” (p04)
	Medications table—no cost or coverage column (5)	“Certainly, for someone who has been at practice for a while, you know what’s covered and what’s not, but for somebody starting off it would be challenging. There are other apps that you can get that information, but it would be nice to have that all in one place for sure. So, cost and covered, yes/no” (p08)
Links	‘Medications’ page—SwitchRx link—links to video, would be better if linked directly to switching tables (2)	“Switching antipsychotics, yeah, but what’s that… okay that’s not what I was looking for. We have this table at work that our pharmacist gave us. This is neat though, but I don’t really want to watch a video. It would be neat if that was just a little info table not a video.” (p10)
	‘Psychiatry consultation ’ page—link to eConsult links to a document describing it, should link to the actual service (1)	“E-consult service yeah, I feel like I would be much more likely to use that than not. Oh, but it takes you to this. Why doesn’t it take you to their website?… But it’s just telling you about… take it to the website where you can log in and then send your consult.” (p05)
Completeness	Suggestions for specific tools, information, or functions that could be added (6)	“It would be good if I could somehow print a handout that has all the websites for them to just go to. So for me to open them and print them all is not really what I want to do right now, but to be able to have a one-page handout that had these websites, these books, these handouts for patients, or that you could maybe check the boxes off and then they would compile on to something and print it off on one page. Because you could say, ‘oh yeah sleep’s an issue for you’… and then ‘here are the books and here are some websites that may be great for you.’ Because I don’t quite know how to give this to them except for what I do now, which is I write some down, or sometimes with their phone take a picture of websites as I open them… it would be great if we could just click and then somehow it knows to print those.” (p06)
***Theme 3: Interactivity of the algorithm and embedded tools***		
Usefulness	Some tools more useful in interactive format (4)	“I would do a PHQ9 and so, oh, that’s nice it pulls it up, but you can’t write on it. Or can you write on it? No you can’t, oh can you, no. I got all excited that you could circle it and then print it off.” (p05)
	Some tools more useful as static PDF format (2)	“the only thing I don’t like… the patient would have to do it on my keyboard, right? Q: Okay so do you think like perhaps just a PDF copy they could fill out? A: Potentially. Presumably we’re gonna go a different way eventually with this, but at the moment that would be the easiest thing. And without the clicky boxes, right, it would have to have a blank.” (p03)
	Would be useful if I input selections and it took me to the sections I needed (3)	“Hmm if I use an algorithm for this specific patient then probably it will be better if… it is just a click… and it gives me (an) automatic score… and (the) score will go to the algorithm and it will bring me to, for example, if it’s ‘yes’ it will go to ‘any suicidal ideation’ automatically” (p17)
Workflow	The algorithm could or should be used flexibly (4)	“with any algorithm there’s a balance between having everything there and having it so inclusive that you can’t work through it in a reasonable amount of time. It looks like this one you can pick and choose what you want, and the stuff is fairly succinct… I think you can use it as much or as little as you want… the algorithm is such that you could click on whatever box you need” (p08)
	May be difficult switching between algorithm and patient chart (1)	“I don’t think it would be an either-or situation by time… the only harm would be integrating with their patient chart and switching back and forth sometimes I suppose” (p20)
***Theme 4: Clarity of presence, purpose, or function of components***		
Layout or organisation	Duplicate boxes containing same information (10)	“Okay we go back and medications and therapy. Is it actually the same information here as what is in here, yeah?” (p17)
Navigation	Clarity of ‘click-ability’ of boxes (10)	“So they’re mild depression, it doesn’t sound like it but, I’m clicking on it (mild depression box) but nothing’s happening.” (p04)
Layout or organisation	Medications table—laid out well (6)	“I like this medication listed in very nice way, written in the side effects on the sides, and pros and cons for each medicine” (p13)
	Medications table – queried the order (6)	“so right now they’re in alphabetical order?… another thing you could do I suppose would be to have it so that you could click different orders. So, you’d have one button say order by amount of anticholinergic effect or order by amount of sedation and then this order would flip around. So, if you’re looking for something with minimal anticholinergic, you would see them first.” (p08)
Understandability	Medications section—queried definitions or meanings (5)	“Now is that to say that it causes insomnia and agitation or that it’s effective for insomnia and agitation?” (p19)
Visibility	‘Switch medication’ and/or 'Augmentation' links not immediately noticed (4)	“I’m not sure if there’s anything on augmenting, like to augment therapy with a second antidepressant, if there’s a section on that that would be really helpful too” (p15)
	‘Print’ and/or ‘Email this page’ functions not immediately noticed (8)	“Now can you print this page out? Patient instruction handout, is there a way to print out those books?… Q: if you go up to the top here A: Oh print” (p10)
Understandability	‘Email this page’ function—queried purpose or how it works (5)	“I don’t understand as much for the psychiatry consultation ‘email this page’, I’m not sure it would link well. I can understand for the education piece… sometimes you actually don’t really want to give anything to them for that (psychiatry) piece. So, when I click on this ‘chronic suicidal risk’ and if I go to email this patient, I’m just curious to see what it’s gonna say. Yeah, so would you send that to your patient?” (p14)
***Theme 5: Navigational challenges and functionality of links***		
Navigation	Easy to find information needed or navigate around the algorithm (4)	“all this information is concentrated on the one algorithm and actually it’s easy to navigate from the one part to another part” (p17)
	Attachments and links opening on current tab over algorithm (8)	“I’ve printed it, I’m gonna close that, gonna make that bigger, that didn’t work though Q: If you just go back here just to Google Chrome A: Did I just totally? Q: Yeah so the PHQ9 opened over this. So you need to click the back button to go on A: Gotcha to go back. I see it doesn’t open up in a new page.… I guess that would be good if it could just print and then I close it and we’re right back to this.” (p06)
	Queries about going back to overall algorithm or previous pages (7)	“Definitely worthwhile chatting to him about psycho education, let’s see what the algorithm says to do for his level of distress. How do we go back here?” (p02)
Links	Suggestion: clicked links could change colour (1)	“This was, I’m not really sure which one I just picked on… I mean in terms of seeing which one I had just picked, because there’s quite a bit, if the links maybe changed to a different colour then I’d know which one I click on back to.” (p16)
	Patient resource link—can ’t access as password protected (1)	“Physical activity as well. Online flip book what’s this? Ha, this book you’re trying to access is password protected.” (p14)

*n* number of participants, *GAD7* General Anxiety Disorder, *PHQ9* Patient Health Questionnaire-9

#### Optimizing content and flow to align with issues faced in practice

Most participants commented that aspects of the content or flow of operations aligned with the approaches they use in practice. However, some noted differences, or highlighted areas where content could more closely align with issues faced in practice. For example, additional content to support treatment of mild depression (including advice on medications to prevent worsening of symptoms) was suggested. Whilst some were enthusiastic about the ‘email this page’ function, which can be used to send content directly to patients, usability was limited due to misalignment with existing patient communication infrastructures. One key issue was raised with the overall algorithm flow diagram itself: participant queried the direct link between the presence of a complex presentation and recommendation for a psychiatry consultation, with some suggesting softening of the language to more closely align with the nuanced clinical judgment that would be made in practice. Some also noted that there are differences in the ‘level’ or nature of complexity of the listed factors, noting that whilst this is the first section where anxiety appears, anxiety is often considered earlier in the diagnostic process and so should appear earlier in the algorithm.

#### Enhancing the most useful algorithm components

Specific algorithm components were commonly referred to as being good features, helpful, or useful, namely: the medications section, the patient education section, and the ‘email this page’ function. Participants emphasised the usefulness of the criteria for choosing an antidepressant, and the table listing dosage guidance and side effects information. Having this information in one place was viewed as a great asset of the algorithm, and helpful for comparing side effects profiles to tailor medication choices to patient preferences. Some participants recommended further columns be added to provide information on the average time to impact, or medication cost/coverage through the provincial drug funding system. Ensuring that the embedded links to additional tools outside the algorithm took users directly to those tools, rather than to descriptions of those tools, was also suggested. In a few instances, suggestions for additional tools, information, or functions were made.

#### Interactivity of the algorithm and embedded tools

Some embedded tools (such as screening/diagnostic questionnaires) were in a static PDF format (needing to be printed, filled out, then scanned back and saved to the patient’s chart), and others were interactive (could be filled out on the computer then saved directly into the chart). Whilst some participants expressed a desire for tools to be interactive, others were happy with the static format. A few participants expected the algorithm to be more interactive or responsive, i.e. taking users through the relevant steps based on their previous responses or inputs, noting that because it is labelled as an algorithm, it should *“choose my path for me”* (p05). Others noted that the algorithm as designed can be used flexibly: users can dip in and out of it during a consultation as they see fit. One participant noted that it may be difficult to interact with the algorithm during a consultation since it involves switching between the algorithm and the patient’s chart.

#### Clarity of presence, purpose, or function of components

Many participants noticed that some algorithm boxes are duplicates which contain the same information (i.e. the patient education, medications, and psychotherapy boxes). Whilst this was not viewed as problematic, it took time to realise this. A bigger issue was the lack of clarity regarding which boxes were ‘clickable’ (where clicking would take users to another page containing more detail), and which were not. There were numerous instances of participants attempting to click an ‘un-clickable’ box and waiting for a new page to load. Specific clarification issues were identified within the medications section. Some participants queried the order the medications were listed in, as the rationale for the existing order was not immediately apparent. Some of these participants also expressed a desire for functionality to change the order, which would support the prioritisation of medications depending on priorities regarding side effects. There were also some issues with understandability of abbreviations and shorthand notation. Some participants did not notice the links to more information on switching or augmenting medications, or the print/email functions, as evidenced by their verbalisations requesting these features when visible on-screen. Finally, some queried the purpose and functioning of the ‘email this page’ function, querying what it sends, what/who the sender is, whether/how to send specific collections of items, and the relative appropriateness of the function on a range of different page types.

#### Navigational challenges and functionality of links

Some participants found it easy to navigate through the algorithm. However, many participants inadvertently closed the algorithm webpage after clicking on and then closing an embedded tool, or a link to an outside resource, not realising that it had opened on the same web browser tab. Some noted it would be better if the tool or resource had opened in a new tab. Others queried how to return to the algorithm after clicking a resource, or how to return to previous pages after moving to different pages. Two pertinent comments were made about embedded links, with one participant noting that clicked links changing colour would help them keep track of what they had done (e.g. patient resources they wanted to check back to), and another identifying a link to a password-protected document.

### Barriers to and enablers of algorithm use in day-to-day practice

We identified important barriers/enablers within ten TDF domains: Knowledge, Social/professional role and identity, Social influences, Intention, Goals, Beliefs about consequences, Memory, attention, and decision processes, Behavioural regulation, Environmental context and resources and Nature of the behaviour. Key belief statements coded to these domains were grouped into five themes, described below. Table 3 presents sample quotes for each theme. Domains not represented in these themes are listed in Additional File 4 with a rationale for exclusion.

**Table 3:** Relevant domains, key belief statements, and sample quotes for five over-arching themes regarding barriers to and enablers of algorithm use in routine practice

Domains	Key belief statements (n)	Sample quotes
***Theme 1: Getting to know the algorithm***		
Knowledge	I’m not familiar with specific tools within the algorithm (barrier) (15)	“I’ve never used the PHQ2. I’m not even sure what that is.” (p16)
	I'm familiar with some of the specific tools within the algorithm (enabler) (7)	“Yeah we were encouraged to use PHQ2 and PHQ9 PHQ2 for initial assessment” (p07)
	I would need to be more familiar with the algorithm to be able to use it (barrier) (9)	“I think to use this you’d just have to go through it a few times before seeing patients so that you understand how it works and read through some of the guidelines so you’re more familiar with them.” (p08)
Environmental context and resources	I would need to invest time in getting to know the algorithm (barrier) (5)	“Like it would take a lot of time to actually be able to use this website well, although I could see the more you use it you might get your favourites and it would be good.” (p10)
	I need to know the algorithm will be kept up-to-date (barrier) (7)	“I think just making sure that the resources are like research-based and I guess there’s some sort of verification process for having them updated? … because there’s nothing worse than giving someone a resource and it’s not, you know… So, I guess knowing that it’s gonna be monitored by somebody at some point.” (p04)
***Theme 2: Alignment with roles and pathways of influence***		
Social/professional role and identity	My professional confidence/experience reduces likelihood of using the algorithm (barrier) (3)	“To a large extent with experience I’m not sure that I need the algorithm.’ (p18)
	The algorithm would be of most benefit to those with less experience (enabler) (6)	“I think probably where I would use it the most would be for new learners… here I can say look, this is a really good tool that you can start to look at in terms of an approach to depression… I had to learn this sort of in a piecemeal fashion, but… this is much more methodical in the approach… sometimes the learners are quite, they really appreciate guidelines.” (p09)
	No role changes would be needed for the algorithm to be taken up (enabler) (7)	“Q: And do you think using the algorithm would require any changes to roles and responsibilities of any of your colleagues here including yourself? A: I don’t think so, no.” (p08)
	Those in various depression care roles could use the algorithm (enabler) (13)	“I think a medical student could use this. A medical resident could certainly use this. A medical doctor could use this. A nurse practitioner could use this too, any prescriber in short… I think like a psychiatry resident could use this. A psychiatrist could use this… I think the nurses could use this. It would be very useful for the nursing team.” (p09)
	Nurses may need support/direction in using components of the algorithm most applicable to them (barrier) (7)	“Well our nurses tend to be kind of worried about stepping into areas where there’s something where they might need a medical directive… so we wouldn’t want them to be feeling like they had to make a diagnosis or come up with a treatment plan or whatever… there may be bits of it that are more applicable to their role than other bits” (p03)
Social influences	My colleagues might influence me to use the algorithm (enabler) (6)	“I guess if other people are saying ‘oh yes, I use that’, I might be like whoa, if they’re using it” (p05)
	Development by known/respected psychiatrist encourages me to use the algorithm (enabler) (4)	“Anything he says I’ll do sure [laugh] that’s why I’m doing it… and he kind of sees it from our perspective of primary care. So it’s always nice to see that.” (p01)
	Extent of uptake with family physicians will determine my (nurse practitioner) use of the algorithm (barrier) (2)	“They’re [the patients] not rostered to me so if the physician’s preference is not to use that, that’s their patient so that would certainly be something that I would have to consider… so typically if a physician says ‘I don’t like to use these particular treatments or for this particular patient I prefer using this’, that’s their patient. Whereas, you know, in a nurse practitioner-led clinic or in other clinics where they are rostered to me, I am their primary provider. That position lies on me.” (p16)
	Residents using the algorithm may encourage uptake (enabler) (2)	“I will probably show the residents this website… and in fact the residents are the best conveyer of change there is… so they might push their other supervisors to use this because they can justify their actions. So it’s the other way around. [laugh]” (p18)
	Team uptake might encourage algorithm use (enabler) (6)	“If everybody is working from the same algorithm then we’re going to have to know the algorithm… it’s gonna be driven by the whole team.” (p09)
***Theme 3: Integration with current ways of working***		
Goals	The algorithm does not conflict with current practice standards (enabler) (7)	“Q: and does it conflict with any do you think? Oh I don’t think so because we’ve been using these tools… like PHQ9 has some suggestion from the mild, moderate and how to manage… it’s congruent so I don’t think it has conflict.” (p07)
	The algorithm adds to current practice standards (enabler) (11)	“It’s a lot more complete. It’s a lot more detailed in terms of giving you guidance and things like that. We don’t really have anything like that… And it’s got local stuff in it which is helpful. We go to Up-to-Date or AMA guides or whatever but they are not necessarily what’s done here. So that’s nice that it’s local.” (p03)
	Using the algorithm may conflict with current practice standards regarding maintaining patient confidentiality (barrier) (3)	“We are absolutely forbidden to email patients in this organization because of privacy concerns…. I mean you’re emailing somebody depression algorithm stuff. Guess what the diagnosis is, yeah.” (p03)
Memory, attention, and decision processes	I may forget to use the algorithm/may need a reminder to use it (barrier) (10)	“Forgetting-wise, it might be for the patients you know really well and… [you’re] in that routine which, sometimes the routine is a bad thing, sometimes you miss things, right, so it’s good to have this in that situation but definitely forgetting to use it in that situation, yeah… Maybe little reminders about the fact that it exists… a couple times/year at our [physician] meetings” (p15)
Environmental context and resources	A slow internet connection may hinder use (barrier) (4)	“I guess the other thing is it depends on how well your Internet is working. Sometimes our Internet gets a bit bogged down here and that may be frustrating but that’s a technical problem.” (p08)
	More integration with the EMR is needed (barrier) (13)	“Well it would be most useful if it was somehow built into our EMR… where you could open this up and it would be saved into the patient’s file… In our electronic record now I can do it [PHQ9] as part of the note… or I can print it off and have it scanned into their file and then write down their score under the diagnosis, and I just find that I wouldn’t use this then if I had to open up the other one anyway.” (p05)
	No additional resources are needed to use the algorithm (enabler) (5)	“I work on a computer so it’s not difficult in that way. It’s not like I work on paper charts and then you have to pull in a computer. And I have printers in my rooms so I could choose any of those handouts and use them. Yeah I don’t think it would need.” (p20)
Behavioural regulation	Saving the algorithm on my computer/browser could encourage me to use it (enabler) (12)	“Well I would probably put it on as a preferred tool on my laptop for sure so it’s accessible all the time.” (p14)
***Theme 4: Contexts for use***		
Intention	I’m motivated to use the algorithm (enabler) (15)	“I think it’s a great idea. I think that I will use it, yeah.” (p08)
	My intention to use the algorithm would depend on the situation (barrier/enabler) (6)	“I would use it as much as I thought it would help me, and in each situation that might be different. In some situations, it might be not required and in other situations, it might be very, very useful.” (p03)
Goals	Using the algorithm could be a priority as it can serve as a consultation guide (enabler) (4)	“I think it would be a pretty high priority because it helps guide. So once you’re used to it it’s gonna be pretty quick and could actually save you time… having everything on one page or one website, not going back and forth.” (p12)
Environmental context and resources	I lack time during a consultation to use the algorithm (barrier) (15)	“If I don’t have a lot of time I’m probably not gonna go through the algorithm but, you know, if I’ve got a longer appointment booked with them and doing a little bit of counselling even, I might bring up the algorithm and discuss some options for them.” (p19)
Memory, attention, and decision processes	I wouldn’t consider using the algorithm in less challenging situations (barrier) (7)	“Well possibly a patient who I’ve been seeing for some time with depression who things are pretty stable. I probably wouldn’t see a reason to open it for that.” (p8)
Social influences	I wouldn't use the algorithm when the patient is very emotional (barrier) (3)	“I think you have to play it by ear with the patient in terms of like if you’re going to lose rapport when you’re using a computer that’s always like it doesn’t matter if it’s an algorithm or taking notes it depends if they come to you and like breaking down in tears I’m never gonna use that for them.” (p20)
Nature of the behaviour^a^	I would be more likely to use the self-management or education resources (8)	“So yeah patient education I think will be where I would go to almost more than anything. If they’re good patient education handouts, because really the diagnosis is usually not that difficult, safety is not that difficult.” (p09)
	I would or do use the algorithm for help with medications (6)	“Most of the time I use it is when I’m going to second line treatment or if I’m switching… they come back and (are) not doing well and so now my question is should I go up on this med? Should I completely switch or should I add something?” (p01)
	I would use the algorithm for help with complex cases of depression (8)	“I think that the cases where things are not going quite the way I think they are, I may go back… I’m sure there’s stuff about follow-up in the refractory to treatment (section) that would have been useful.” (p3)
	I could or do use the algorithm with patients (8)	“And it’s a good thing to go through with patients actually.” (p19)
	I wouldn’t use the algorithm with patients (4)	“It’s a little bit much that I wouldn’t show it to patients per se” (p20)
	I would or do use the algorithm before or after seeing a patient (5)	“A: I would use it between patients or before seeing a patient… Q: And would that be perhaps to think about how you would move forward maybe? A: Yeah, not in a concrete way because you haven’t interviewed the patient yet but to give you some ideas of what I might be missing and where it could go with that patient.” (p02)
***Theme 5: Anticipated benefits and concerns about patient communication***		
Social/professional role and identity	Using the algorithm may interfere with my therapeutic role (barrier) (3)	“I mean one of the therapeutic things that we do is to listen to people, right? And so if they’re talking and they’re distressed etc. then, you know, as a family doctor that’s one of my major tools in my toolbox is to listen and hear what they’re saying and help them reflect back on it all, that sort of thing. So that’s probably why I wouldn’t be enthusiastic about clicking the screen a lot during a patient encounter.” (p03)
Beliefs about consequences	Using the algorithm would improve care (enabler) (13)	“I mean clearly there’s some good information in there. It might improve depression care, I mean often we cut corners and skip things and forget things. So, it might help to make things more complete… it might help patients to get the kind of care they need when they need it.” (p03)
	Algorithm use may help standardize depression care within and beyond primary care (enabler) (10)	“And I would probably push my other colleagues, physicians or nurse practitioners to use it to so we have the same plan… so we think about the patient the same way. So if there’s not a lot of variance between each clinician, that would make it safer… within here but also within the Champlain LHIN. So if all psychiatrists are using the same thing, they will understand if we send a referral for consultation that we did try everything else before because we’re following all the same guidelines. So therefore it should make it smoother when they get our consultation I would think.” (p14)
	I don’t foresee any negative consequences of using the algorithm (enabler) (5)	“I have no negative outcomes that I can imagine.” (p18)
	Algorithm use could have negative impacts if not used with clinical judgment (barrier) (4)	“Q: Can you foresee any potential harms or negative impacts that could come from using it? A: Total reliance or ‘concrete thinking’ use of the algorithm without application of clinical reasoning for the specific scenario, because nothing’s going to perfectly fit into any one of those boxes.” (p19)
	Using the algorithm improves my access to resources for supporting patients (enabler) (12)	“I think the centralization of good evidence-based resources is what would drive me to use this… Often you’ll get handouts from like patient-specific websites and then you have to go to the CPS and get the medications and so this is nice and centralized.” (p20)
	Algorithm use may improve patient involvement in or satisfaction with care (enabler) (3)	“I just like knowing the side effects [of medications]. To be able to show people this and review some of it and then they could sort of play a more active role. And if we’re trying to decide between two [medications], what they might like.” (p06)
	Algorithm use may reduce patient confidence (barrier) (2)	“Maybe if patient’s not happy if, you know, ‘hey, why you still using textbook to guide the care? [laugh] Maybe at that point… they think that you are not very professional, you don’t know anything.” (p07)
	Algorithm use could improve appointment time management/reduce workload (enabler) (7)	“So time management, it would be super helpful because you’re right there with all the good resources. Yeah even access to medication information is so available… so it could reduce your workload. If you did have the capacity in a clinic where they could pre-screen with a PHQ9 like they could come to you and like even if you included the like medical type questions in some capacity that a nurse could screen for. They could enter your office with kind of a moderate depression and then it would just be like confirming these symptoms and then yeah moving on from there. So yeah it could ease the workload.’ (p20)
	Algorithm use may increase screen time/reduce attention on the patient (barrier) (5)	“Increasingly we’re sort of spending more time at the computers and screens so as long as we still try to maintain that human contact… that’s probably the biggest risk is that people start clicking through this and even though it’s quite user friendly, if you start doing that in front of patients it might turn them off.” (p02)
	Algorithm use would not negatively impact relationships with patients (enabler) (2)	“I’m a big believer in contemporaneous charting using a computer with a patient. Many of my colleagues don’t support that, but so considering I am doing that, this [using the algorithm] would be an adjunct…. Patients understand people use computers. They want you to be able to document effectively. They want you to provide them with information that’s accessible. And I believe that even though I’m a thousand, 50-mistake a minute typist—I wasn’t brought up on computers—I’m continuously using the computer while I’m seeing a patient and I’m sure, I’m positive that that doesn’t interfere with my relationship with a patient.” (p18)

*n* number of participants, *AMA* American Medical Association, *CPS* Compendium of Pharmaceuticals and Specialties, *EMR* Electronic Medical Record, *LHIN* Local Health Integration Network, *PHQ2/PHQ9* Patient Health Questionnaire-2/9

^a^Belief statements coded to the Nature of the behaviour domain were not categorised as barriers or enablers as this domain serves as a description of the behaviour (in terms of who/when/what/where/how) rather than an influencing factor

#### Getting to know the algorithm

Most participants were unfamiliar with the algorithm and at least some of the embedded tools. Many acknowledged that they would need to get to know the algorithm before using it directly with patients or consulting it to help support depression care, and find time in their already hectic work or home contexts to learn about algorithm content, establish which components might be most helpful or applicable, and develop the procedural knowledge required to use those components. It was also noted that this algorithm is more ‘involved’ than some others that they use, and that it would help if this was made clear to users up-front. Some participants raised concerns about how up-to-date the algorithm is with respect to the best available evidence regarding depression care, and emphasised that they would need to know more about how the algorithm would be kept up-to-date and by whom.

#### Alignment with roles and pathways of influence

Most participants agreed that the algorithm could be used by range of primary care professionals. Some noted that because nurses are only involved in specific aspects of depression care, guidance regarding the algorithm components most applicable to their role could encourage uptake in nurses. Despite the algorithm being designed to assist all practitioners regardless of experience level, a few of the more experienced physicians explicitly noted that they did not perceive a need for the algorithm, and that it would be more helpful to less experienced practitioners. Some participants noted that uptake amongst their colleagues or their team more generally could encourage them to use the algorithm. Two nurse practitioners highlighted that the patients they saw were rostered to the physicians they worked with, and the physicians would ultimately influence the extent to which nurse practitioners would use the algorithm. However, one nurse practitioner noted that this would not apply to nurse practitioner-led clinics. Two participants noted that uptake with residents could be a powerful influence on more senior colleagues through the demonstration of how useful or helpful the algorithm is. Finally, a few participants commented that they knew and respected the psychiatrist who led algorithm development, which would encourage them to use it.

#### Integration with current ways of working

Some participants noted that they did not see any conflicts between the algorithm and other recommendations or evidence-based standards they worked to. However, some noted concerns regarding use of the algorithm and their imperative to maintain patient confidentiality (particularly in relation to the email function and leaving the algorithm open/visible on screen). Many agreed that the algorithm adds to existing guidelines/standards, and could replace other resources currently used. Some noted that they had access to all the resources they needed to use the algorithm (e.g. a computer, an internet connection, printing facilities); however, practice internet connections can be slow, which may hinder use. Despite the potential ease of integration into practice, participants thought that they might forget to use the algorithm during a consultation where it might have helped them. Simple solutions such as saving the algorithm as a favourite in their preferred browser or as an icon on their desktop were described as potentially helpful strategies for integration into practice routines. Most participants felt that integration into their electronic medical record (EMR) would be the key strategy for helping them remember to use it and for enabling them to build it into their workflows. Participants mentioned different possible levels of integration (e.g. an electronic reminder which would link out to the algorithm, or full integration whereby any screening tools or questionnaires completed using the algorithm would be automatically saved into patient charts).

#### Contexts for use

Most participants intended or wanted to use the algorithm, with some noting that they could see using the algorithm becoming a priority. However, most noted that time constraints were a barrier to use during a consultation. Motivation varied depending on specific algorithm components and factors such as consultation/patient type and perceived need for help. Participants were motivated to use the patient resources and medications sections. Some participants did not feel the need to use the algorithm is situations which could be described as less challenging, i.e. with patients they know well, with stable patients, or with relatively straightforward cases. Participants would be more likely to use the algorithm in more complex situations. Some described this more generally in terms of getting stuck/not knowing what to do next, whilst others gave specific examples (e.g. an initially selected medication has been unsuccessful and guidance on switching or augmenting medications is needed). For some participants, algorithm use would depend on the emotional state of the patients: they would not want to use it when the patient was very emotional and there was the potential to lose rapport or harm the communicative aspects of care. There were differences of opinion regarding using the algorithm in front of patients. As an alternative, some participants described using the algorithm before or after as a source of ideas.

#### Anticipated benefits and concerns about patient communication

Participants noted numerous potential benefits of using the algorithm. Many noted that use would increase their access to a broader range of resources for supporting patients, whilst also centralising these resources. Whilst some cautioned that the algorithm could have negative impacts if applied without the co-application of clinical judgment, most agreed that incorporating the algorithm into their practice will help them help their patients and ultimately improve care. Many described the potential for standardisation or streamlining of care, noting the potential for the algorithm to improve consistency in the care that patients receive when they see different professionals within a practice, and also when referred out (e.g. to psychiatry services). Relatedly, some noted that once teams were familiar with the algorithm, using it could result in time savings/reduced workloads, achieved through the ease of access to centralised resources, and streamlining processes within care teams (e.g. nurses completing screening assessments and physicians focusing on treatment approaches). Participants also discussed the potential for negative consequences regarding patient communication. Two noted that visible algorithm use may reduce patient confidence in their clinical expertise. Some were concerned about increased screen time and reduced attention on the patient, with some specifically highlighting a tension between algorithm use and their therapeutic role in mental health-focused consultations where active listening and providing support are crucial. However, two participants felt that using the algorithm in a consultation would not negatively impact relationships, whilst three noted that discussing parts of the algorithm with patients could help to increase patient involvement and satisfaction.

## Discussion

This study investigated factors influencing the usability and routine uptake of an online algorithm supporting primary care professionals in caring for people with depression. Participants were enthusiastic about using the algorithm, found it easy to use, and viewed specific components as particularly helpful. Participants thought it could be used by those in different roles, could see it replacing other tools due to its centralisation of resources covering the care pathway, and noted the potential to influence standardisation of care. However, there are opportunities to improve alignment with workflows, enhance usefulness, optimize interactivity, enhance clarity, and mitigate challenges with navigation and links. Participants also emphasised their need to get to know algorithm content before incorporating it into their care, identified those in specific roles whose uptake would influence others, acknowledged that they might forget to use the algorithm when it could have been helpful, noted concerns about increased screen time, and felt that integration into their EMR would support routine use. By combining usability testing methods with a behavioural science framework, this study has provided insights to inform both modifications that could be made to algorithm content and functionality, and broader strategies to support implementation of the algorithm in routine practice.

### Implications for optimizing algorithm structure, function, and content

Some changes have already been made to the algorithm to address the usability issues identified. To more closely mirror the diagnostic process, discussion of anxiety has been removed from a later step in the algorithm and integrated earlier, and the GAD-7 for anxiety screening has been added [32]. In response to concerns about the direct link between a complex presentation (described as frequent) and recommendation for a psychiatry consultation (described as not always appropriate or required), algorithm language has been modified to prompt *consideration* of a referral. The appearance of all boxes has been amended to more clearly visually indicate ‘clickability’, with clickable boxes now looking like buttons. Where feasible, embedded links now take users directly to the relevant service website, rather than to a document describing the service. The email function and links to information on medication switching and augmentation are now more noticeable.

Other potential avenues for optimization require additional resources. These include changes to the medications table, viewed as one of the most useful components of the algorithm. Usefulness could be enhanced by providing information on the average time to impact for each medication, adding a column reporting cost/health plan coverage, and allowing users to change the order of medication presentation depending on patient/clinical priorities for minimising specific side effects. Such changes would help to address important information needs and subsequently support shared decision making [33]. A 2½ minute video has been added to guide use of the medications section as it is currently presented.

In some instances, the potential pros and cons of making changes need to be considered. Some participants wanted more guidance for mild depression, and there was specific interest in symptom-focused medication. Guidelines do not recommend medication as a first-line treatment for mild depression [9], and other protocols developed to support depression care have been viewed positively for encouraging the treatment of mild depression without medication [34]. Therefore, such changes would need to be carefully thought through so as not to risk encouraging over-medication and focus specifically on symptom management. Other potential changes need to be considered from a feasibility perspective. Providing different versions of all embedded tools (i.e. static pdf and interactive formats) requires resources to gather such tools and depends on their availability and requirements for use, since these tools were developed by others. Developing a version of the algorithm which is fully interactive or responds to user inputs would involve significant work to change functionality. In addition, such functionality is less aligned with intended use. The algorithm was designed to be a general resource that can be used flexibly, as opposed to a step-by-step decision support tool, with many participants appreciating this aspect of the design.

### Implications for an implementation strategy

The findings provide an evidence base to inform the selection of strategies for encouraging algorithm uptake. The identified lack of knowledge about the algorithm suggests that strategies to raise awareness combined with focused education and training could be a good place to start. Awareness-raising can involve presentations at conferences/meetings, and emailing information about the algorithm to relevant society member lists [34]. Education and training could be operationalised in multiple ways depending on resources available and intended reach. Locally-focused activities could involve interactive workshops embedded within team meetings, whilst instructional videos could be developed and disseminated to reach a broader audience. It may be important to embed instructions on how to use specific components of the algorithm, offer demonstrations of use, and provide opportunities for practice or rehearsal [35]. Various formats could be considered, such as written descriptions, videos of mock interactions with patients, live observations, written scenarios to support individual practice, or role play activities. Since we identified context-specific intention to use the algorithm, demonstrations and opportunities for practice could incorporate various examples of situations it can be used in (e.g. using the algorithm with a patient who is stable; when a patient is very upset, reviewing the algorithm after the patient has left). Whilst this may support algorithm uptake, the range of other factors influencing use indicates that additional strategies would also be necessary, such as focusing on social influence processes, clarifying how those in different roles can use the algorithm in accordance with their scope of practice, and/or embedding reminders at the point of intended use.

### Combining usability testing and behavioural theory-informed barriers/enablers assessment

We combined two methodological approaches to provide insights on two distinct but interrelated concepts: factors impacting tool usability, and factors impacting tool use in routine practice. This has resulted in a more comprehensive investigation than would have been achieved if we had only included one component or the other. Assessing barriers to and enablers of implementation is a key component of many implementation process models [15, 36, 37] and is often done using behavioural theory-informed frameworks which focus on identifying factors influencing the target behaviour in routine practice. In instances such as this where a new tool is being implemented, our findings indicate that supplementing this traditional approach with an element of usability testing can provide additional insights. Whilst the findings related to usability allow us to propose changes to the tool itself, the findings from behaviour change theory-informed interviews allow us to propose strategies to encourage uptake of the tool, both of which should ultimately support integration of the tool in day-to-day practice.

Whilst we did not conduct a systematic assessment of conceptual overlap in our coding and theme generation between the two data sources, our coding indicates the usability categories and TDF domains for which some overlap can occur. These are: *Workflow* and *Environmental context and resources* (lack of time to use the algorithm); *Workflow* and *Nature of the behaviour* (discussion of use in specific situations); *Content* and *Knowledge* (familiarity with the resources included in the algorithm); *Usefulness* and *Beliefs about consequences* (comments on the increased access to resources); and *Hardware & software* and *Environmental context and resources* (slow internet connection hampering use). Since these issues relate to use in everyday practice with likely solutions being strategies to support uptake rather than changes to the algorithm, we felt they were best represented in the TDF section. Other usability issues (*Understandability*, *Completeness*, *Layout & organization*, *Visibility*, *Navigation*, and *Links*) did not overlap with content coded to the TDF domains, which further support the added value of usability testing.

**Fig. 2:**
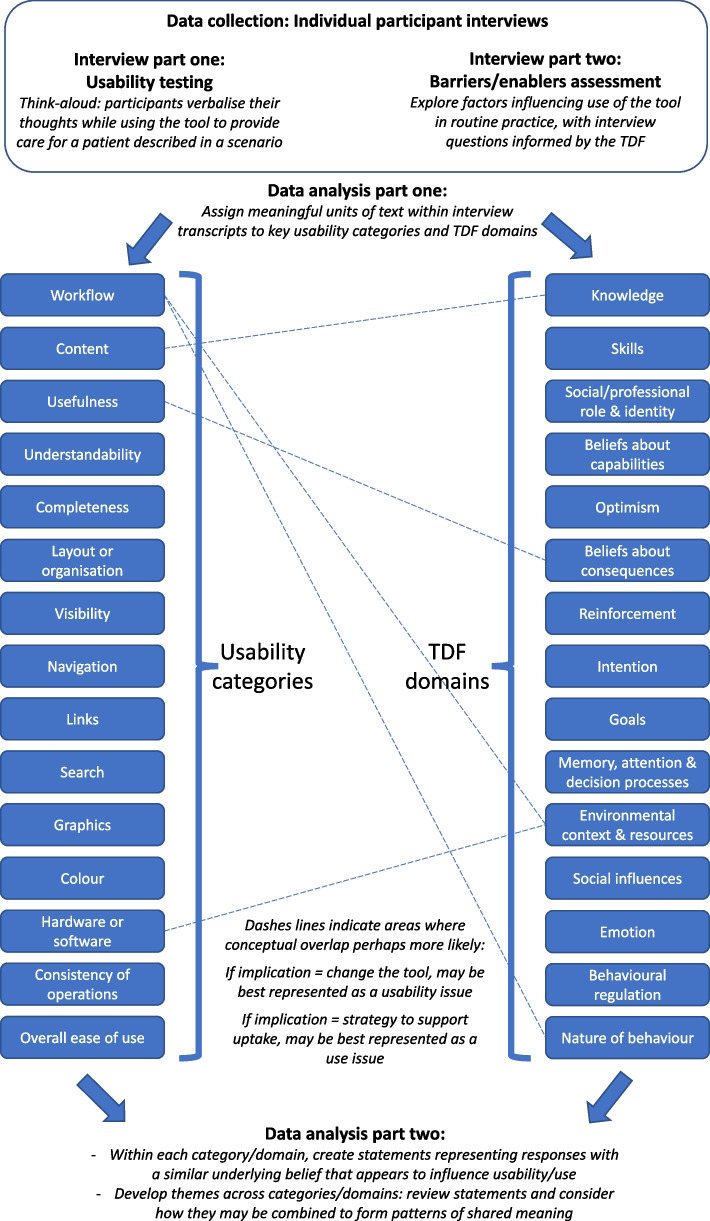
Combining theory-based barriers assessments and usability testing to inform optimization and implementation of clinical tools: methods guide

Other researchers have looked at barriers to implementation and usefulness and usability issues in the same study as part of tool development [38, 39]. However, these studies have some key differences with ours. Anderson and colleagues [38] recruited one group of clinicians to firstly watch a video demonstration of the tool and then provide feedback on implementation barriers and enablers, and a different group of clinicians to think aloud while using the tool in a simulated environment. Coleman and colleagues [39] randomised clinicians to either a think-aloud interview to identify specific usability issues or a focus group to identify general usability issues, for which they provide implementation as an example. Whilst these previous approaches provide useful examples, a unique contribution of our work is that we have built our approach on pre-existing frameworks which outline a range of usability issues [27–31] and factors influencing behaviour [12, 13], which may help to increase comprehensiveness. Our work may therefore provide a useful example for those conducting behaviour change theory-based barriers assessments on how they can integrate different methodological approaches to potentially broaden the insights they obtain. In Fig. 2, we provide a methods guide for others who may wish to supplement barriers assessments with usability testing.

### Strengths and limitations

Through an innovative combination of methodological approaches, this project has identified usability issues with, and barriers to, uptake of an evidence-informed clinical tool. This work has already guided efforts to improve the tool and can now be used to inform an implementation strategy designed to enhance uptake. Our approach may be informative for those looking to implement similar tools in healthcare contexts. All participants practiced in interdisciplinary team-based environments and so our findings may not be generalizable to settings involving different models of care. Whilst we used written scenarios during think-aloud testing, it is possible that realism may be enhanced and/or different usability issues may be identified using different methods such as involving patient actors [38] or using different forms of usability testing altogether such as near-live clinical simulations [29]. However, participant responses during the think-aloud tasks did seem to represent a description of their approach had the patient been real, and participants did not appear to have any difficulties with identifying usability issues during this portion of the interview. Whilst usability testing forms an important part of User-Centred Design approaches, which are increasingly being advocated for in implementation science [19], we did not have the capacity to conduct a User-Centred Design study, wherein multiple rounds of testing is required to use these approaches effectively [19]. Whilst others have integrated behaviour change theory into multi-round User-Centred Design approaches [40], our approach is built on a multi-theory framework which identifies a broad range of behavioural influences and so may increase the comprehensiveness of barriers identified whilst also providing an example of a method for enhancing the informativeness of behaviour change theory-based barriers investigations.

## Conclusions

This study identified a range of usability issues and barriers to use of an online algorithm designed to support primary care professionals in caring for people with depression. The findings have informed some initial changes to the algorithm designed to enhance usability, and will also be reflected on to inform subsequent updates. The identified barriers to use indicate that implementation strategies focusing on awareness-raising about algorithm existence, education and training, social influence processes, and changing the physical environment may be best placed to enhance uptake. This study serves as an example of methods for combining two methodological approaches and integrating existing frameworks to broaden the insights obtained from behaviour change theory-based barriers assessments which involve implementation of a new tool.

## Supplementary Information

Supplementary Material 1.

Supplementary Material 2.

Supplementary Material 3.

Supplementary Material 4.

## Data Availability

Data excerpts may be available upon reasonable request to the corresponding author.

## Abbreviations

EMR: Electronic Medical Record
TDF: Theoretical Domains Framework

## References

[1] Lam RW, McIntosh D, Wang J, Enns MW, Kolivakis T, Michalak EE, et al. Canadian Network for Mood and Anxiety Treatments (CANMAT) 2016 clinical guidelines for the management of adults with major depressive disorder. Section 1. Disease burden and principles of care. Can J Psychiatry. 2016;61:510–23. 27486151 10.1177/0706743716659416 PMC4994789

[2] Community Preventive Services Task Force. Recommendation from the community preventive services task force for use of collaborative care for the management of depressive disorders. Am J Prev Med. 2012;42:521–4. 22516494 10.1016/j.amepre.2012.01.010

[3] Kates N, Mach M. Chronic disease management for depression in primary care: a summary of the current literature and implications for practice. Can J Psychiatry. 2007;52:77–85. 17375862 10.1177/070674370705200202

[4] MacMillan HL, Patterson CJS, Wathen CN, Feightner JW, Bessette P, Elford RW, et al. Screening for depression in primary care: recommendation statement from the Canadian Task Force on Preventive Health Care. CMAJ. 2005;172:33–5. 15632399 10.1503/cmaj.1030823 PMC543939

[5] Miedema B, Tatemichi S, Thomas-Maclean R, Stoppard J. Barriers to treating depression in the family physician’s office. Can J Commun Ment Health. 2004;23:37–46. 15920881 10.7870/cjcmh-2004-0003

[6] Thombs BD, Coyne JC, Cuijpers P, de Jonge P, Gilbody S, Ioannidis JPA, et al. Rethinking recommendations for screening for depression in primary care. CMAJ. 2012;184:413–8. 21930744 10.1503/cmaj.111035 PMC3291670

[7] Whitebird RR, Solberg LI, Margolis KL, Asche SE, Trangle MA, Wineman AP. Barriers to improving primary care of depression: Perspectives of medical group leaders. Qual Health Res. 2013;23:805–14. 23515301 10.1177/1049732313482399

[8] Mental Health Algorithms. Welcome to the Ottawa Depression Algorithm. https://ottawadepressionalgorithm.ca/en/start. Accessed 1 Dec 2022.

[9] Lam RW, Kennedy SH, Parikh SV, MacQueen GM, Milev RV, Ravindran AV. Canadian Network for Mood and Anxiety Treatments (CANMAT) 2016 clinical guidelines for the management of adults with major depressive disorder Introduction and methods. Can J Psychiatry. 2016;61:506–9. 27486152 10.1177/0706743716659061 PMC4994787

[10] Eccles MP, Mittman BS. Welcome to Implementation Science. Implement Sci. 2006;1:1.

[11] Davidoff F, Dixon-Woods M, Leviton L, Michie S. Demystifying theory and its use in improvement. BMJ Qual Saf. 2015;24:228–38. 25616279 10.1136/bmjqs-2014-003627 PMC4345989

[12] Michie S, Johnston M, Abraham C, Lawton R, Parker D, Walker A, et al. Making psychological theory useful for implementing evidence based practice: a consensus approach. Qual Saf Health Care. 2005;14:26–33. 15692000 10.1136/qshc.2004.011155 PMC1743963

[13] Cane J, O’Connor D, Michie S. Validation of the theoretical domains framework for use in behaviour change and implementation research. Implement Sci. 2012;7:37. 22530986 10.1186/1748-5908-7-37 PMC3483008

[14] Francis JJ, O’Connor D, Curran J. Theories of behaviour change synthesised into a set of theoretical groupings: introducing a thematic series on the theoretical domains framework. Implement Sci. 2012;7:35. 22531601 10.1186/1748-5908-7-35 PMC3444902

[15] French SD, Green SE, O’Connor DA, McKenzie JE, Francis JJ, Michie S, et al. Developing theory-informed behaviour change interventions to implement evidence into practice: a systematic approach using the Theoretical Domains Framework. Implement Sci. 2012;7:38. 22531013 10.1186/1748-5908-7-38 PMC3443064

[16] Witteman HO, Dansokho SC, Colquhoun H, Coulter A, Dugas M, Fagerlin A, et al. User-centered design and the development of patient decision aids: protocol for a systematic review. Syst Rev. 2015;4:11. 25623074 10.1186/2046-4053-4-11 PMC4328638

[17] Norman D. The Design of Everyday Things: Revised and. Expanded. New York, USA: Basic Books; 2013.

[18] Abras C, Maloney-Krichmar D, Preece J. User-centered design. In: Bainbridge W, editor. Encyclopedia of Human-Computer Interaction. Thousand Oaks, CA, USA: SAGE Publications Ltd; 2004. p. 445–56.

[19] Haines ER, Dopp A, Lyon AR, Witteman HO, Bender M, Vaisson G, et al. Harmonizing evidence-based practice, implementation context, and implementation strategies with user-centered design: a case example in young adult cancer care. Implement Sci Commun. 2021;2:45. 33902748 10.1186/s43058-021-00147-4 PMC8077816

[20] Kiran T, Moineddin R, Kopp A, Frymire E, Glazier RH. Emergency department use and enrollment in a medical home providing after-hours care. Ann Fam Med. 2018;16:419–27. 30201638 10.1370/afm.2291 PMC6130993

[21] Francis JJ, Johnston M, Robertson C, Glidewell L, Entwistle V, Eccles MP, et al. What is an adequate sample size? Operationalising data saturation for theory-based interview studies. Psychol Health. 2010;25:1229–45. 20204937 10.1080/08870440903194015

[22] Ericsson KA, Simon HA. Protocol Analysis: Verbal Reports as Data. Rev. Cambridge, MA, US: The MIT Press; 1993.

[23] van Someren MW, Barnard YF, Sandberg JAC. The Think Aloud Method: a Practical Approach to Modelling Cognitive Processes. London, England: Academic Press; 1994.

[24] Atkins L, Francis J, Islam R, O’Connor D, Patey A, Ivers N, et al. A guide to using the Theoretical Domains Framework of behaviour change to investigate implementation problems. Implement Sci. 2017;12:77. 28637486 10.1186/s13012-017-0605-9 PMC5480145

[25] Braun V, Clarke V. Can I use TA? Should I use TA? Should I not use TA? Comparing reflexive thematic analysis and other pattern-based qualitative analytic approaches. Couns Psychother Res. 2020;21:37–47.

[26] Braun V, Clarke V. One size fits all? What counts as quality practice in (reflexive) thematic analysis? Qual Res Psychol. 2020;18:328–52.

[27] Kushniruk AW, Patel VL, Cimino JJ. Usability testing in medical informatics: cognitive approaches to evaluation of information systems and user interfaces. Proc AMIA Annu Fall Symp. 1997:218–22. https://pmc.ncbi.nlm.nih.gov/articles/PMC2233486/. PMC2233486 9357620

[28] Kushniruk AW, Patel VL. Cognitive and usability engineering methods for the evaluation of clinical information systems. J Biomed Inform. 2004;37:56–76. 15016386 10.1016/j.jbi.2004.01.003

[29] Li AC, Kannry JL, Kushniruk A, Chrimes D, McGinn TG, Edonyabo D, et al. Integrating usability testing and think-aloud protocol analysis with “near-live” clinical simulations in evaluating clinical decision support. Int J Med Inform. 2012;81:761–72. 22456088 10.1016/j.ijmedinf.2012.02.009

[30] U. S. Department of Health and Human Services. Research-based Web Design & Usability Guidelines. Washington, DC: U.S. Government Printing Office; 2006.

[31] US General Services Administration. Usability.gov. 2013. https://www.usability.gov/index.html. Accessed 1 Dec 2022.

[32] Spitzer RL, Kroenke K, Williams JBW, Löwe B. A brief measure for assessing Generalized Anxiety Disorder: The GAD-7. Arch Intern Med. 2006;166:1092–7. 16717171 10.1001/archinte.166.10.1092

[33] Uhler LM, Pérez Figueroa RE, Dickson M, McCullagh L, Kushniruk A, Monkman H, et al. InformedTogether: Usability evaluation of a web-based decision aid to facilitate shared advance care planning for severe chronic obstructive pulmonary disease. JMIR Hum Factors. 2015;2:e2. 27025896 10.2196/humanfactors.3842 PMC4797670

[34] Roberge P, Fournier L, Brouillet H, Delorme A, Beaucage C, Côté R, et al. A provincial adaptation of clinical practice guidelines for depression in primary care: a case illustration of the ADAPTE method. J Eval Clin Pract. 2015;21:1190–8. 26083732 10.1111/jep.12404

[35] Michie S, Richardson M, Johnston M, Abraham C, Francis J, Hardeman W, et al. The Behavior Change Technique Taxonomy (v1) of 93 hierarchically clustered techniques: Building an international consensus for the reporting of behavior change interventions. Ann Behav Med. 2013;46:81–95. 23512568 10.1007/s12160-013-9486-6

[36] Graham ID, Logan J, Harrison MB, Straus SE, Tetroe J, Caswell W, et al. Lost in knowledge translation: Time for a map? J Contin Educ Health Prof. 2006;26:13–24. 16557505 10.1002/chp.47

[37] Meyers DC, Durlak JA, Wandersman A. The quality implementation framework: a synthesis of critical steps in the implementation process. Am J Community Psychol. 2012;50:462–80. 22644083 10.1007/s10464-012-9522-x

[38] Anderson JA, Godwin KM, Saleem JJ, Russell S, Robinson JJ, Kimmel B. Accessibility, usability, and usefulness of a web-based clinical decision support tool to enhance provider–patient communication around Self-management TO Prevent (STOP) Stroke. Health Informatics J. 2014;20:261–74. 24352597 10.1177/1460458213493195

[39] Coleman S, Nixon J, Keen J, Muir D, Wilson L, McGinnis E, et al. Using cognitive pre-testing methods in the development of a new evidenced-based pressure ulcer risk assessment instrument. BMC Med Res Methodol. 2016;16:158. 27852237 10.1186/s12874-016-0257-5 PMC5112672

[40] Lawani MA, Turgeon Y, Côté L, Légaré F, Witteman HO, Morin M, et al. User-centered and theory-based design of a professional training program on shared decision-making with older adults living with neurocognitive disorders: a mixed-methods study. BMC Med Inform Decis Mak. 2021;21:59. 33596874 10.1186/s12911-021-01396-y PMC7888116

