# Supplementary material for: Combining theory and usability testing to inform optimization and implementation of an online primary care depression management tool
Source: BMC Med Inform Decis Mak. 2025 Jan 15;25:25. doi: 10.1186/s12911-024-02733-7 (PMC11734350; doi:10.1186/s12911-024-02733-7)
Supplement: Supplementary file 1 — Supplementary Material 1. [file 12911_2024_2733_MOESM1_ESM.docx]

**Combining theory and usability testing to inform optimization and implementation of an online primary care depression management tool**

**Additional File 1: Interview guide**

**Introduction to the algorithm**

I’ll start with a very brief introduction to the algorithm, which is loaded here on the laptop. So it’s a clinical pathway designed to be used by primary care providers in the treatment of adult depression in your office, at the point of care. It is not intended either to replace your clinical judgment or to establish a protocol for all patients. Rather, it can be used to help you assess, diagnosis and manage your patient. Should you decide to refer your patient, it has information and links to local resources.

PLAY INTRO/INSTRUCTIONAL VIDEO ON SITE (1.11 MINS)

Any questions about that?

**User-testing (INCLUDE ONLY IF APPROPRIATE GIVEN PARTICIPANT’S PROFESSIONAL ROLE)**

I’m going to give you a scenario which describes a patient presenting to a consultation with symptoms of depression. I’d like you to imagine this patient presenting to you, and that you are going in and using the algorithm to help you decide what to do, in conjunction with your own clinical judgment and the steps you would normally take in your own practice.

We are interested in what you think about as you are going through and using the algorithm, so I am going to ask you to ‘think aloud’ as you do this. What I mean by this is that I want you to tell me everything that you are thinking while you are making your decisions. I don't want you to try to plan out what you say or try to explain to me or justify what you are saying. Just act as if you are alone in the room speaking to yourself. If you are silent for any long period of time I will ask you to ‘keep thinking out loud’. My role is just to remind you to keep going and not to intervene in your thoughts.

Does this make sense?

Here’s the scenario along with some additional information that may become helpful as you progress through the algorithm (HAND OUT SHEETS). If you would ask the patient about or investigate something and I haven’t given you any information on it, please assume it’s not an issue. Please now read the patient details aloud and then go to the algorithm and start to say all your thoughts out loud while working through the algorithm.

***Prompts (if needed): Keep going; keep thinking aloud; go on…; keep thinking out loud***

**(STRAYING FROM ALGORITHM PATH: ‘WELL IF YOU DID USE THE ALGORITHM…’)**

**(RE-EMPHASISE IF NEEDED: ALGORITHM NOT INTENDED TO REPLACE CLINICAL JUDGMENT)**

**(NOTE ISSUES WHICH WANT TO PROBE FURTHER IN TDF QUESTIONS: DON’T ASK NOW AS DISRUPTS THINK-ALOUD)**

Scenario phase 1: initial contact: content to reflect mild depression

*A patient in his/her mid-30s has come to your office describing trouble sleeping at night but excessive sleepiness during that day, and loss of appetite, over the past month. He/she has come to see you because he/she has noticed that this is affecting his/her concentration at work, and he/she is worried about losing his/her job as a result. When you ask him/her about his/her mood, he/she describes “feeling down” most of the time, and that he/she is losing interest in his/her career, which was something he/she used to enjoy. He/she is carrying on with his/her regular social activities but doesn’t have much energy most of the time. He/she is not on any medication at the moment and has no other medical conditions.*

‘Concerns regarding mood?’ box: Two-question screen/ PHQ-2/ PHQ-9

NMc response: Two-question screen: yes to both; PHQ-2: score 3; PHQ-9: score 8 (i.e. symptoms suggestive of mild depression)

‘Confirm diagnosis of depression’ box: Assess/ confirm diagnosis of depression (PHQ-9/ rule out contributory medical conditions/ rule out main psychiatric differential diagnoses/ confirm DSM-5 criteria met)

NMc response: Assess: ‘no’ where appropriate (i.e. no abuse or neglect, no history of depression)/ Confirm diagnosis of depression: PHQ-9: score 8/ no indications for contributory medical conditions or main psychiatric differential diagnoses/ DSM-5 criteria met (presence of symptoms A1, A2, A4, A6, A8, and criteria B-E fulfilled (symptoms cause clinically significant distress or impairment in social, occupational, or other important areas of functioning; The episode is not due to the effects of a substance or to a medical condition; The occurrence is not better explained by schizoaffective disorder, schizophrenia, schizophreniform disorder, delusional disorder, or other specified and unspecified schizophrenia spectrum and other psychotic disorders; There has never been a manic episode or a hypomanic episode)) (i.e. confirm diagnosis of depression)

**‘Any suicidal ideation?’ Not a click-able box**

**NMc response: no recurrent thoughts of death, no recurrent suicidal ideation, without a specific plan, no suicide attempt, no suicide plan: basically, no suicidal thoughts or acts (i.e. no suicidal ideation)**

‘Complex presentation?’ box: Assess for factors listed which, if present, indicate that patient’s condition is complex

NMc response: no suicidal/homicidal risk; no significant dysfunction; no possible bipolar disorder; no substance abuse; no anxiety disorder; no psychosis; no personality disorder; no ADHD; not elderly; no diagnostic uncertainty; refractory to treatment not applicable (first presentation with symptoms of depression therefore no treatment as yet) (i.e. no complex presentation)

‘Mild depression?’ Not a clickable box: may need prompted to go here/ reminder of mild depression diagnosis/ reminder of PHQ-9 score of 8

‘Patient Education’ box: Patient Instructions Handout/ Handouts for Patients/ Books/ Websites/ For Family Members and Friends; Looking for information in other languages

‘Supported Self-Management (SSM)’ box: self-management tools

Scenario phase 2: follow-up contact 1: content to reflect progression to moderate depression

*The patient has come to your office for a follow-up visit two weeks later. He/she is still having difficulty sleeping and still has a low appetite. He/she has increasingly been taking time off work due to having very low energy levels nearly every day. He/she is no longer able to carry on with his/her regular social activities. You re-administer the PHQ-9, and his/her score is 16.*

‘Moderate depression?’ Not a clickable box: may need prompted to go here/ confirmation of moderate depression diagnosis/ reminder of PHQ-9 score of 16

‘Patient Education’ box: Patient Instructions Handout/ Handouts for Patients/ Books/ Websites/ For Family Members and Friends; Looking for information in other languages

‘Psychotherapy’ and/or ‘Medications’ boxes

Psychotherapy: CANMAT 2016 guideline recommendations/ Handout for patients/ Handout for community resources

Medications: Table of antidepressant medications (may click through into ‘Choosing an antidepressant medication’, list of key considerations)

**(PROMPT TO REVIEW MEDICATIONS SECTION OF ALGORITHM IF NOT DONE AS PART OF CASE)**

Great, thanks for talking me through your thoughts.

1. Based on this initial experience of using the algorithm, what do you think worked well, and not so well?

Thank you. For the rest of the interview, I have some specific questions that focus on you using the algorithm. In answering these questions, I want you to think about the following specific activity:

**Using the Ottawa Depression Algorithm to help you screen for, diagnose and manage depression in your primary care setting.**

**If at any point you would like me to repeat this activity, please let me know.**

Some questions may seem repetitive, but please bear with me as they do try to get at slightly different things that may influence your use of the algorithm.

**(BOLD = KEY QUESTION FOR EACH DOMAIN, ASK ONLY THESE IF SHORT ON TIME)**

| PROMPTS if detail limited  Think about other algorithms/care pathways: those that have been implemented successfully in your practice, as well as those which were not taken up  (e.g. for mental health issues, for other chronic conditions like heart disease, asthma, diabetes)   - What was different about the ones taken up? - What helped them be integrated into workflows? - What hindered those not integrated?   To what extent could you see this applying to the Ottawa Depression Algorithm?  Think about screening for, diagnosing, and managing depression: are there any aspects of that process which would need special consideration when implementing an algorithm/care pathway? |
| --- |

**Knowledge**

**(WHAT DO THEY KNOW, AND HOW DOES THAT INFLUENCE WHAT THEY DO?)**

1. **Do you use any guidelines or local policies for screening, diagnosing and managing depression in primary care?**
   - **PROMPT: If yes, which guidelines/policies, and what do they recommend (e.g. CANMAT, APA, DSM, ICD)? If no, what do you use to guide your practice?**
2. **Will using the algorithm add to any guidelines you currently use, or other processes or procedures that you usually follow in your practice? How/why?**
3. **Will using the algorithm conflict with any of these guidelines/processes/procedures? How/why?**

(WOULD THIS INFLUENCE WHETHER OR NOT YOU USE THE ALGORITHM?)

**Skills**

**(WHAT DO THEY KNOW ABOUT HOW THEY SHOULD BE DOING SOMETHING**

**AND HOW DOES THAT INFLUENCE WHETHER THEY DO IT OR NOT?)**

1. **What experience or expertise do you think you will need to use the algorithm to help you screen for, diagnose and manage depression?**
   - **PROMPT: Interpersonal skills/rapport with patients?**
   - **PROMPT: experience or expertise needed to use the algorithm to administer, score, and stratify according to the screening questionnaires (i.e., the PHQ-2 and PHQ-9)?**
   - **PROMPT: experience or expertise needed to use the algorithm to provide education/self-management support?**
   - **PROMPT: experience or expertise needed to use the algorithm to appropriately prescribe medication to treat depression?**
   - **PROMPT: experience or expertise needed to use the algorithm to appropriately refer for psychiatry consultation?**
2. Do you think that you have the necessary experience or expertise to use the algorithm? If not, what could help?

(WOULD THIS INFLUENCE WHETHER OR NOT YOU USE THE ALGORITHM?)

**Social/ Professional Role and Identity**

**(HOW DOES WHO THEY ARE AS A HCP INFLUENCE WHETHER THEY DO SOMETHING OR NOT?)**

1. Would you see it as part of your job/responsibility to use the algorithm to help you screen for, diagnose and manage depression in your primary care setting? Are there others who should be involved? Who should be involved, why, and how?
2. **Would using the algorithm require any changes to the roles and responsibilities of any of your team members, including yourself?**
   - **PROMPT: change to your role?**
   - **PROMPT: change to resident/trainee role & learning?**
   - **PROMPT: change to nurses/nurse practitioner roles?**

**If so,**

**(a) what changes would be required**

**(b) how could these be implemented, and**

**(c) would you anticipate any problems arising?**

(WOULD THIS INFLUENCE WHETHER OR NOT YOU USE THE ALGORITHM?)

**Beliefs about Capabilities**

**(DO THEY THINK THAT THEY CAN DO WHAT THEY SHOULD DO**

**AND HOW DOES THAT INFLUENCE WHETHER THEY DO IT OR NOT?)**

1. **How easy or difficult would it be for you to use the algorithm to help you screen for, diagnose and manage depression in your primary care setting?**

**What would make it easier?**

- - **PROMPT: What makes it easy to use? Why?**
  - **PROMPT: What makes it difficult to use? Why?**
  - **PROMPT: (give examples of things that may make it easier if needed, and then ask to elaborate: patient factors, colleagues, process of care, equipment, the practice)**

1. How confident do you feel in your ability to use the algorithm? What makes you feel less confident/what would make you feel more confident?
2. How much power or control do you think you have over your ability to use the algorithm in your setting? What things would influence your use that are beyond your control? What would increase your control?

(WOULD THIS INFLUENCE WHETHER OR NOT YOU USE THE ALGORITHM?)

**Optimism**

**(HOW DOES WHETHER THEY ARE OPTIMISTIC/PESSIMISTIC INFLUENCE WHAT THEY DO?)**

1. **How optimistic are you that using the algorithm will help you screen for, diagnose and manage depression in your primary care setting? Why?**
   - **PROMPT: level of optimism high/low?**

(WOULD THIS INFLUENCE WHETHER OR NOT YOU USE THE ALGORITHM?)

**Beliefs about consequences**

**(WHAT ARE THE GOOD AND BAD THINGS THAT CAN HAPPEN FROM WHAT THEY DO**

**AND HOW DOES THAT INFLUENCE WHETHER THEY’LL DO IT IN THE FUTURE?)**

1. **What do you think would be the benefits or positive impacts of using the algorithm?**
   - **PROMPT: For yourself, your patients, your colleagues, and your primary care setting**
   - **PROMPT: improve patient health, improve patient access to resources?**
   - **PROMPT: improve your practice/knowledge/skills?**
   - **PROMPT: help you not forget education & self-management support important**
   - **PROMPT: colleagues/setting: improve consistency/standardisation of practice?**
   - **PROMPT: impact on workload?**
2. **Are there any harms or negative impacts that you think would occur from using the algorithm?**
   - **PROMPT: For yourself, your patients, your colleagues, and your primary care setting**
   - **PROMPT: screen-time during consultation vs. human/eye contact**
   - **PROMPT: patient perception of number/box in flowchart**
   - **PROMPT: impact on workload?**
3. Do the potential benefits of using the algorithm outweigh the potential harms? If not, what could help achieve this?

(WOULD THIS INFLUENCE WHETHER OR NOT YOU USE THE ALGORITHM?)

**Reinforcement**

**(HOW HAVE THEIR EXPERIENCES (GOOD AND BAD) OF DOING IT IN THE PAST INFLUENCE WHETHER OR NOT THEY DO IT?)**

1. **Are there any incentives or rewarding experiences that would encourage you to use the algorithm?**
   - **PROMPT: (e.g. satisfaction knowing that you’re using evidence-based practice, doing everything you can to ensure good patient outcomes, rewarding part of job, seeing patient progress, avoiding referral and successful management in setting, things that could make it fun to use?)**
2. Can you foresee any sanctions that may be associated with using the algorithm?

(WOULD THIS INFLUENCE WHETHER OR NOT YOU USE THE ALGORITHM?)

**Intention**

**(HOW DOES HOW INCLINED THEY ARE TO DO SOMETHING INFLUENCE WHETHER THEY WILL DO IT?)**

1. **To what extent do you want to use the algorithm to help you screen for, diagnose and manage depression in your primary care setting?**
   - **PROMPT: In what situations may you find yourself *more* motivated to use it? Why?**
   - **PROMPT: In what situations may you find yourself *less* motivated to use it? Why?**
   - **PROMPT: complex cases (ask to define), switching medications, refractory to treatment/non-responders, screening vs. diagnosis vs. management**

(WOULD THIS INFLUENCE WHETHER OR NOT YOU USE THE ALGORITHM?)

**Goals**

**(HOW IMPORTANT IS WHAT THEY DO AND DOES THAT INFLUENCE WHETHER OR NOT THEY DO IT?**

**WHAT STANDARDS ARE THEY TRYING TO REACH, HOW DOES THAT INFLUENCE WHETHER OR NOT THEY DO IT?)**

1. Would using the algorithm be something that you feel you would want or need to do? What would drive that?
2. **Thinking about everything you have to do with a patient, how much of a priority would it be for you to use the algorithm compared to other priorities that you may have?**
   - **PROMPT: why would/wouldn’t it be a priority?**

(WOULD THIS INFLUENCE WHETHER OR NOT YOU USE THE ALGORITHM?)

**Memory, attention and decision processes**

**(HOW DOES THEIR FORGETFULNESS OR REMEMBERING TO DO IT INFLUENCE WHETHER OR NOT THEY DO IT?**

**HOW DOES THEIR ABILITY TO FOCUS ON THE BEHAVIOUR IINFLUENCE WHETHER OR NOT THEY DO IT?**

**HOW DO THE DECISIONS THEY MAKE ABOUT THE BEHAVIOUR INFLUENCE WHETHER THEY DO IT OR NOT?)**

1. Could you see using the algorithm becoming an automatic or routine part of your work, or would it be something you would need to stop and take time to think about?
2. In what situations would you decide to use the algorithm? Why?
3. In what situations might you decide not to use the algorithm? Why? What would you do instead?
4. **In what situations could you see yourself forgetting to use the algorithm? Why?**

**What could help you to integrate the use of the algorithm into your routine or habits?**

- - **PROMPT: (e.g. when clinic is running behind, when depression is not primary reason for consultation, not having the website open): what could help you remember?**

(WOULD THIS INFLUENCE WHETHER OR NOT YOU USE THE ALGORITHM?)

**Environmental Context and Resources**

**(WHAT ARE THE THINGS IN THEIR ENVIRONMENT THAT INFLUENCE WHAT THEY DO**

**AND HOW DO THEY INFLUENCE? (NOT JUST PHYSICAL STUFF, BUT ACCESS TO OTHER PROFESSIONALS)**

1. How would you use the algorithm to help you screen for, diagnose and manage depression in your primary care setting?
   - PROMPT: On a computer? By memory? In patient consultations? As a learning tool?
2. What aspects of your practice setting would influence your use of the algorithm?
3. Are there competing tasks/demands or time constraints that could interfere with your use of the algorithm? What are these? What could help overcome this?
4. **Is there anything in your work environment that might influence your likelihood of using the algorithm?**

**Are there any additional resources you would need in order to use the algorithm?**

- - **PROMPT: physical set-up/environment, IT aspects, access to other resources, foresee any logistical problems?**
  - **PROMPT: to what extent are additional resources needed available?**

(WOULD THIS INFLUENCE WHETHER OR NOT YOU USE THE ALGORITHM?)

**Social Influences**

**(WHAT DO OTHERS THINK OF WHAT THEY DO?**

**WHO ARE THEY AND HOW DOES THAT INFLUENCE WHAT THEY DO?)**

1. **Who would influence your likelihood of using the algorithm, and how?**
   - **PROMPT: colleagues; residents/trainees; patients; others?**
2. How might the views or opinions of others affect your use of the algorithm?
3. Do you think your practice colleagues would use the algorithm? What about providers in other practices?

(WOULD THIS INFLUENCE WHETHER OR NOT YOU USE THE ALGORITHM?)

**Emotion**

**(HOW DO THEY FEEL ABOUT WHAT THEY DO AND DO THOSE FEELINGS INFLUENCE WHAT THEY DO?)**

This may be a bit of an odd question, as it’s about your emotions, but bear with me, as it is important that we talk about whether your feelings about the algorithm might influence how you use it.

1. Would you have any worries or concerns about using the algorithm?
2. **How do you *feel* about using the algorithm (when you think of using this algorithm, what sorts of emotions come to mind)?**
   - **PROMPT: anxiety, stress, positive emotion?**
   - **PROMPT: things that could make it fun to use?**

(WOULD THIS INFLUENCE WHETHER OR NOT YOU USE THE ALGORITHM?)

**Behavioural Regulation**

**(WHAT DO THEY THINK WOULD HELP / WHAT STRATEGIES HAVE HELPED THEM DO WHAT YOU SHOULD DO?**

**WHAT STRATEGIES ARE ALREADY IN PLACE TO HELP THEM DO WHAT THEY SHOULD DO?)**

1. What strategies or supports or ways of working are already in place in your practice setting that would help you use the algorithm?
2. **If you wanted to implement changes in your own practice setting to encourage use of the algorithm, what would be some ways to do this?**
   - **PROMPT: introduce supports/ strategies/ new ways of working (e.g. multidisciplinary)?**
   - **PROMPT: introductory session at team meeting/lunch? More involvement of nurses?**

(WOULD THIS INFLUENCE WHETHER OR NOT YOU USE THE ALGORITHM?)

1. Thinking of everything we have just discussed, what do you think are the most important factors that would influence your use of the Ottawa Depression Algorithm?
2. The algorithm interface is currently web/text: what are your thoughts on developing a ‘chatbot’ version which you could verbally interact with (e.g. ask a specific question and it would tell you the answer)?
3. Is there anything else you’d like to expand on?

Those are all the questions I have for you. I appreciate the time and insight that you’ve given me today. Is there anything else related to this topic you would like to talk about that we haven’t covered?

STOP RECORDING NOW
