# Supplementary material for: Combining theory and usability testing to inform optimization and implementation of an online primary care depression management tool
Source: BMC Med Inform Decis Mak. 2025 Jan 15;25:25. doi: 10.1186/s12911-024-02733-7 (PMC11734350; doi:10.1186/s12911-024-02733-7)
Supplement: Supplementary file 2 — Supplementary Material 2. [file 12911_2024_2733_MOESM2_ESM.docx]

**Combining theory and usability testing to inform optimization and implementation of an online primary care depression management tool**

**Additional File 2: Written patient scenario for usability testing**

*(initial consultation - sheet provided to participant)*

1

A patient in {his/her} mid-30s has come to your office describing trouble sleeping at night but excessive sleepiness during that day, and loss of appetite, over the past month. {He/she} has come to see you because {he/she} has noticed that this is affecting {his/her} concentration at work, and {he/she} is worried about losing {his/her} job as a result. When you ask {him/her} about {his/her} mood, {he/she} describes “feeling down” most of the time, and that {he/she} is losing interest in {his/her} career, which was something {he/she} used to enjoy. {He/she} is carrying on with {his/her} regular social activities but doesn’t have much energy most of the time. {He/she} is not on any medication at the moment and has no other medical conditions.

*(follow-up visit - sheet provided to participant)*

2

The patient has come to your office for a follow-up visit two weeks later. {He/she} is still having difficulty sleeping and still has a low appetite. {He/she} has increasingly been taking time off work due to having very low energy levels nearly every day. {He/she} is no longer able to carry on with {his/her} regular social activities. You re-administer the PHQ-9, and {his/her} score is 16.

*(additional information - sheet provided to participant)*

No suicidal ideation

Two-question screen: patient answers ‘yes’ to both questions

PHQ-2 score is 3

PHQ-9 score is 8

No indications for contributory medical conditions or main psychiatric differential diagnoses

DSM-5 criteria: presence of symptoms A1, A2, A4, A6, A8, and criteria B-E fulfilled

No anxiety

*(If you would ask the patient about or investigate something for which there is no information here, please assume it is not an issue for this patient)*
