# Supplementary material for: Combining theory and usability testing to inform optimization and implementation of an online primary care depression management tool
Source: BMC Med Inform Decis Mak. 2025 Jan 15;25:25. doi: 10.1186/s12911-024-02733-7 (PMC11734350; doi:10.1186/s12911-024-02733-7)
Supplement: Supplementary file 3 — Supplementary Material 3. [file 12911_2024_2733_MOESM3_ESM.docx]

**Combining theory and usability testing to inform optimization and implementation of an online primary care depression management tool**

**Additional File 3: Analysis codebook**

**User testing**

Coding instructions:

- Code all relevant text into the usability code (i.e. provide context to demonstrate how the text is associated with a code). Note to double coder: only code the highlighted text, but use the broader context text to inform the coding. Can code un-highlighted text if deemed relevant.
- Code highlighted text to multiple codes if necessary
- Do not code general comments related to how participants decide to diagnose/treat/manage the patient described in the scenario unless it specifically relates to usability of the algorithm
- Consider also coding to TDF domains if relevant (see coding instructions below)
- Note that responses may be hypothetical since we are focusing on uptake and use of the algorithm going forward
- Note that transcripts in NVIVO are supplemented with annotations informed by video data (e.g. where participants are discussion a specific part of the algorithm but do not name it, the name has been added to aid interpretation): please review annotations to assist coding
- It may also be helpful to review the algorithm itself, to assist interpretation of the transcripts: <https://www.ottawadepressionalgorithm.ca/en/start> (NOTE: the algorithm has been updated since the interview took place and so some features may now differ from descriptions in transcripts)

| **Code** | **Definition** | **Instructions** | **Examples** |
| --- | --- | --- | --- |
| Workflow | General order and sequence of tasks and activities involved in a patient encounter, which explicitly link to using the algorithm | Code comments relating to:  Practicalities of using the algorithm during a consultation/hypothetical consultation/as part of the process of care for depression  Integration of algorithm into current workflows  Integration of algorithm into current IT systems/electronic medical records  Include suggestions for improvement related to workflow (for example, modifications that could enhance integration with current workflows)  If explicit link not made to integration with workflow and comment on the algorithm is more general, code to ‘content’ | “Anything that you came across that you thought wasn’t quite right or didn’t quite work or?  R: Well I think with any algorithm like that there’s a balance between having everything there and having it so inclusive that you can’t work through it in a reasonable amount of time…So it looks like this one you can pick and choose what you want and the stuff is fairly succinct. You know if you’re looking at a resource like Up-to-Date or something there’s so much information you don’t know where to start or even if you do start you get bogged down. So this looks like it’s a reasonable amount of information.” FP5  “R: So this is a form what I can print out but again if I’m sitting in this electronic charting is extra unnecessary work to print it out, and to fill it out, to fill it up and then scanning it back to the system. I think if it we can open in a separate window where we can, we can just click, click, click and it put as a score it will be much more beneficial.” NP7 |
| Content | The information included on the site and within the algorithm | Code comments relating to:  The content of the information provided by the algorithm  Specific algorithm features  Can include highlighting of similarities/ differences between current practice and algorithm content/flow (but if explicit link made to integration of the algorithm into workflows, code to ‘workflow’)  General positive/negative comments about specific aspects of content (but if explicit link made to usefulness, code to ‘usefulness’)  Include suggestions for improvement related to specific content or features | “R: Okay I’m gonna pick Cipralex for her. Okay 5 in the morning, up to 10, okay so what is this telling me over here? Well I would probably do that start her on 5 and increase her to 10. And then reassess her which is what I usually do and then yeah that’s generally my approach. If she’s doing really well I would wait and keep her on same dose and then if she was improving I might continue the same or I might increase depending on the situation. No improvement I would definitely increase and reassess and then definitely go all the way up to 20 if they weren’t improving before switching. Okay so I like that.” FP7  “R: I like the quality of the resources. A lot of them are resources that I’ve already used” R1  “So again with that I’d like the result table right as soon as I open it or maybe that could just be a separate thing like PHQ9 result table.” FP4 |
| Usefulness | Extent that the algorithm (and information provided by it) is perceived as helpful during clinical decision-making and care delivery | Code comments relating to:  Usefulness/helpfulness of algorithm in general  Specific parts of the algorithm that will be helpful in practice/not  Include suggestions for improvement related to usefulness  If explicit link not made to usefulness/helpfulness and comment on the algorithm is more general, code to ‘content’ | “R: Switching antipsychotics yeah no this is yeah but what’s that  I: Yeah that’s not so this is linking out to another.  R: Okay that’s not what I was looking for. We have this table at work that our, our pharmacist gave us. Oh this is neat though but I don’t really want to watch a video.  It would be neat if that was just a little info table not a video. Guidelines okay.  I: Yeah there may be I think there is I think if you have like an account with them you can see it or something. I think the table just pops up.  R: Yeah hmm I don’t think that’s good. That would that’s annoying. Oh register now.  I: Yeah you need to register.  R: [laugh] Boo I don’t like that.  I: Another thing [laugh]  R: But yeah because definitely having there’s a chart similar to this which has antidepressants along the side and along the top and then it tells you to taper and what for and you can just go directly one day off one day on. That would be useful to have on here I would say.” FP7  “And in terms of medications I often talk about the different classes and what fits the patient’s profile the most in terms of SSRI or SNRI or other ones. In terms of their priorities for side effects, weight gain, versus sleep, versus sexual side effects etc. so to actually be able to go through the, this myself or with the patient would be really helpful actually because some patients come to me saying that they absolutely don’t want to gain weight, they absolutely don’t want the sexual side effects so it’s nice to show them this chart actually. Otherwise it’s more of an abstract conversation so this is really nice for a lot of my patients. It’s really good actually. Perfect.” FP9 |
| Understandability | Extent to which the algorithm and its content is comprehendible | Code comments relating to:  How understandable the display (graphics and/or text) is  Understandability of icons, required computer operations, and system messages  Comprehension of acronyms/abbreviations  Comprehension of content/terminology  Include suggestions for improvement related to understandability | “I’m trying to understand these stepped care so I’m wondering why it says stepped care throughout because in my mind this is more shared care here when you get psychiatry involved.  But I’m probably missing something in the terminology. Stepped care to me means giving the right intervention at the right time. So I guess it is stepped care because you’re yeah because you’re, you’re increasing your interventions as the patients need them.  We, we referred to these phases as shared care once we get psychiatry involved here. That’s, that’s more the local lingo and I wonder if it might be worth sort of highlighting that?  That level 3 and 4 is shared care.” FP2  “Insomnia 1+ so I’m assuming you’ve got a scale of what this means, the 1+? No? Okay so that would be helpful. [laugh]” NP6 |
| Completeness | Extent to which the algorithm includes all required steps & information | Code comments related to:  Specific instances/areas where information/content which is additional/complementary to what is there is recommended  Volume of information provided (Too much? Too little?)  Include suggestions for improvement related to completeness | “I think it’s quite, you know, it’s very I don’t know how do I say comprehensive yeah.” NP3  “Do you have any like you have elderly written here but do have like the Geriatric Depression Scale or anything?  I: Mm I don’t know if that’s in there but that’s if missing a good do you think a good tool that would be  R: We have an elder care program. I know one thing that we use tablets when patients come in they fill out certain forms and Geriatric Depression Scale is a common one that they put on there.” NP4 |
| Layout or organisation | Extent to which: information is clearly organized; all necessary information is available without slowing the user with unneeded information; content facilitates scanning, and enables quick understanding; excessive scrolling is avoided | Code comments related to:  Clarity in organisation of information, including ordering and location  Layout/organisation of overall algorithm, plus individual components  Include suggestions for improvement related to layout or screen organisation | “How easy or difficult do you think it is to use the algorithm to screen for, diagnose and manage depression? And is there anything that might make it easier to use?  R: You might want to put the PHQ questions right in the algorithm because it’s just 2 questions so that might avoid one click.  Hmm that’s all. I don’t think, I don’t think there’s room for the 9 PHQ, PHQ9 questions. But the 2 questions I have to remind myself what they are so it’s probably mood and what are they. Even when I click I have to actually look at look down and see if I can find them. Where am I so it’s depressed mood and anhedonia.  Okay so that might need to be highlighted even here maybe somehow, you know right away up here.  The 2 standardized ways of asking it, mm hmm.” FP2  “in reality this complex presentation probably happens like before confirming the diagnosis of depression actually because this is ruling out other things. So questions like were you hearing voices or especially with bi-polar have you had any episodes where you’re feeling really high or spent a lot of money [26:06] _______ etc. So that would probably have come earlier before confirming diagnosis that’s a question in mind.” R2  “I: But people have mentioned they would like to reorder them on the basis of these columns so they could, you know, if this was important they’d want those medications to be on top and so on.  R: Yeah, yeah oh so if you said okay so this person is really worried about their sexual health not being impacted you’d click on that and it pre orders it automatically?  So that would be nice.  Or based on studies saying that they’re most effective like, you know, a lot of people often say Cipralex and Zoloft are the #1 SSRI that people that we should try so it seems weird that Celexa is there because it’s still not alphabetical. So that bugs me. [laugh]” FP7 |
| Visibility | Extent to which an image, text, or message is noticed or attended to | Code comments related to:  Visibility/prominence of specific items  Include suggestions for improvement related to visibility  Size of items and impact on visibility  Queries about features which are present but query suggests they have not been noticed | “I: Yeah there’s I don’t know if you noticed as well at the top here there’s this email if you click this box, this bit here it basically allows you to select leaflets and then you can email directly to the patient.  R: That’s really I’m sorry I didn’t notice that that’s really great.  I: That’s okay  R: Instead of me printing this and you can spare the paper and so what we just need is email the patient wonderful.  I: Yeah so you just put their email and then it comes from it doesn’t come from your email it comes from like a genetic email address so it wouldn’t  R: So this way it’s not gonna  I: because I know there’s some issues with confidentiality and things they have mentioned in other practices so.  R: Yeah okay so it’s not gonna show my own private email?  I: No  R: Wonderful that’s really good. Thanks for that I didn’t know that.  I: Yeah so maybe yeah most people don’t notice that so I think it just needs to be a bit more prominent but it’s there.  R: Yeah great, great yeah” FP8  “R: The only thing that’s missing here is switching.  I: I think if you click on back on the medications table, right up there yeah so it links you out to it’s not part of the algorithm it’s (talking over)  R: Oh switch medications oh yeah I’m familiar with switch Rx but that’s, that’s helpful I’m glad it’s there.” FP10 |
| Navigation | Methods used to find information within the algorithm/site | Code comments related to:  Basic navigation/moving through the algorithm  Ease or difficulty in finding the desired information  Ease or difficulty of moving through the website or finding something  Queries related to where to go, how to go forward, how to go back  Attempts to click aspects of the algorithm that are not ‘click-able’ (don’t navigate anywhere)  Include suggestions for improvement related to navigation | “I’m assuming I just press the x to get out of the PHQ9?  I: I think you have to click the yeah if you press x it will like close the whole site.  R: Okay  I: So if you press the back the arrow here it’ll go back, yeah.  R: Okay, okay.” NP6  “but this button doesn’t work and you just enlarge.  I: That’s the no that’s not a clickable box so the boxes that are clickable have the bold.  R: Oh that’s not obvious.  I: Yeah you’re not the first to point that out. [laugh]  R: Yeah that’s, that’s not obvious. I’d probably put something more like dots so a dotted border that makes it really different.  Because to me that just looks like it’s been highlighted already.  Whereas this one doesn’t, doesn’t stand out as not being clickable.” FP10 |
| Links | Hypertext links which cause a new page to load (page may be part of the algorithm, or may be link to an external site) | Code comments related to:  Link labels  Consistency between link labels and subsequent page linked to  Appropriateness/usefulness of external sites linked to  Problems with links/broken links  Include suggestions for improvement related to links | “R: Physical activity as well. Online flip book what’s this? Ha this book you’re trying to access is password protected.  I: I’ve not seen anyone click on that yet so, yeah.  R: Maybe I’m too curious but it was there.” NP5  “R: The only thing that’s missing here is switching.  I: I think if you click on back on the medications table, right up there yeah so it links you out to it’s not part of the algorithm it’s (talking over)  R: Oh switch medications oh yeah I’m familiar with switch Rx but that’s, that’s helpful I’m glad it’s there.” FP10 |
| Search | The site’s search capability | Code comments related to:  Location of search option; Functioning of search option; Usefulness of search results  Include suggestions for improvement related to search | The search function is too slow |
| Graphics | Graphics, images, animation, video, and audio | Code comments related to:  Graphics/images/animation/video/ audio specifically  Include suggestions for improvement related to graphics | This image could be made clearer |
| Colour | Use of colour in the algorithm/website | Code comments related to:  Explicit mention of the use of colours  Include suggestions for improvement related to colour | “I’m trying to understand the colours here. So, of course, high suicide risk is red. No it doesn’t bother me the colours work.” FP2 |
| Hardware or Software | Any constraints imposed on users by hardware, software, and speed of connection to the Internet. This includes issues with operating systems and web browsers, and screen resolution. | Code comments related to:  Issues with hardware; issues with software; connection speed; operating system; web browser; screen resolution  Include suggestions for improvement related to hardware or software | “And so I’m just gonna play this introductory video here, hopefully it plays.  R: Thinking it might be dicey in this room we’ll see. [laugh]  R: We seem to have paused that’s probably the Internet.  I: It might be this  R: Yeah because it’s not it’s not it’s still in play mode it’s.” FP3 |
| Consistency of operations | Consistency in how the same or similar actions are completed across different parts of the site | Code comments related to:  Consistency of operations/actions cross the algorithm  Include suggestions for improvement related to consistency of operations | “and this one brings us back to the algorithm. That button should be on many more of the screens [laugh]” FP10 |
| Overall ease of use | Perceived effectiveness, efficiency, and ease-of-use of the algorithm/site | Code comments related to:  How easy the algorithm is to use generally  Overall judgments of user-friendliness  Include suggestions for improvement related to overall ease of use | “R: It’s very user, user-friendly” NP4 |

References used to inform user-testing component of codebook:

- <https://www.usability.gov/how-to-and-tools/methods/reporting-usability-test-results.html>
- <https://www.usability.gov/how-to-and-tools/resources/templates/report-template-usability-test-long.html>
- <https://www.usability.gov/sites/default/files/documents/guidelines_book.pdf>
- Li AC, Kannry JL, Kushniruk A, Chrimes D, McGinn TG, Edonyabo D, Mann DM: Integrating usability testing and think-aloud protocol analysis with "near-live" clinical simulations in evaluating clinical decision support. Int J Med Inform 2012, 81:761-72.
- Kushniruk AW, Patel VL. Cognitive and usability engineering methods for the evaluation of clinical information systems. J Biomed Inform. 2004 Feb;37(1):56-76.
- Kushniruk AW, Patel VL, Cimino JJ. Usability testing in medical informatics: cognitive approaches to evaluation of information systems and user interfaces. Proc AMIA Annu Fall Symp. 1997:218-22.

**TDF questions**

TACT-Specified Behaviour*

| Action: | Using the Ottawa Depression Algorithm to help you screen for, diagnose and manage depression |
| --- | --- |
| Target: | Primary care professionals |
| Time: | ‘going forward’ |
| Context: | in your primary care setting |

*One administrator was interviewed: the relevant behaviour focused on was: **Implementing the Ottawa Depression Algorithm to help providers screen for, diagnose, and manage depression in your primary care setting**

Coding instructions:

- The framework used for data analysis is a combination of 14-Domain Theoretical Domains Framework 2 (TDF2; Cane et al., 2012) with the addition of the domain ‘nature of the behaviour’ from the 12-Domain Theoretical Domains Framework (TDF1; Michie et al., 2005)
- Code quotes into domains (Definitions of constructs and domains are included below). Note to double coder: the double coding is at the domain level.
- Code all responses (both positive and negative).
- Code all relevant text into each domain (i.e. provide context to demonstrate how the text is associated with a domain). Note to double coder: only code the highlighted text, but use the broader context text to inform the coding. Can code un-highlighted text if deemed relevant.
- Please use the “Instructions” columns to supplement the description of the domains and constructs for this context. Also see the instructions column for information regarding text that could be justifiably coded into multiple domains. Can code highlighted text to multiple domains if necessary.
- Code “No” answers along with the corresponding question into the appropriate domains.
- Note that responses are often hypothetical since we are focusing on uptake and use of the algorithm going forward

| **Domain** | **Constructs** | **Instructions** | **Examples** |
| --- | --- | --- | --- |
| **1. Knowledge**  *An awareness of the existence of something*  *What do they know and how does that influence what they do? (AP/JP)* | Knowledge (including knowledge of condition/scientific rationale): *An awareness of the existence of something* | Consider coding to this domain comments on:  Awareness of evidence related to screening for/ diagnosing/managing depression  Awareness of guidelines/resources/local policies for screening for/diagnosing/managing depression (consider also coding at ‘Goals’)  Awareness of algorithm or algorithm content  Complementarity/conflict between guidelines/resources/local policies and the algorithm (consider also coding at ‘Goals’)  How algorithm adds to/does not add to current guidelines/resources/local policies (consider also coding at ‘goals’)    Procedural knowledge: knowing how to screen for/diagnose/manage depression  Procedural knowledge: knowing how to use the algorithm or its components  Practice standards (also consider coding at goals)  Inappropriate coding to this domain:  Discussion of who uses/will use the algorithm (Social Professional Role and ID)  Discussion of how easy/difficult using the algorithm, is/will be (Beliefs About Capabilities)  What they normally do currently regarding use of the algorithm– code to ‘Nature of the Behaviour’ and ‘Social/Professional Role and Identity’ instead  What they normally do currently regarding screening/diagnosing/managing depression– code to ‘Nature of the Behaviour’ instead | “I’m not familiar with the tool so I right now I have to read everything as I’m talking to you and find out where everything is.” NP5  “I’ve never used the PHQ2. I’m not even sure what that is.” NP6  “R: Guidelines or local policies so the guideline that influences us a lot about the screening thing is Canadian Taskforce on Preventive Health Care” FP3  “And do you think using from what you’ve seen of the algorithm will using it add to these guidelines and things that you’re currently using?  R: I, I find it will like I think it’s got a centralized—it’s different from the DSM. The DSM is only helpful for diagnosis and frankly I don’t look at it for depression because we’ve pretty much been taught to memorize it.” FP6 |
|  | Procedural knowledge: *Knowing how to do something* |  |  |
|  | Knowledge of task environment: *Knowledge of the social and material context in which a task is undertaken.* |  |  |
| **2. Skills**  *An ability or proficiency acquired through practice*  *What do they know about how they should be doing something and how does that influence whether they do it or not? (AP/JP)* | Skills: *An ability or proficiency acquired through training and/or practice* | Consider coding to this domain comments on:  Specific experience or expertise or skills needed to use the algorithm generally, or to:  A) administer, score, and stratify according to the screening questionnaires (i.e., the PHQ-2 and PHQ-9)  B) provide education/self-management support  C) appropriately prescribe medication to treat depression  D) appropriately refer for psychiatry consultation  E) use any other components of the algorithm  Training or skills development needed to use the algorithm  How the relationship/rapport between health care professional and patient may facilitate/impede the use of the algorithm (Interpersonal Skills)  Inappropriate coding to this domain:  Ease or difficulty of using the algorithm: code to ‘Beliefs about Capabilities’ instead  What they normally do currently regarding use of the algorithm– code to ‘Nature of the Behaviour’ and ‘Social/Professional Role and Identity’ instead  What they normally do currently regarding screening/diagnosing/managing depression– code to ‘Nature of the Behaviour’ instead | “we didn’t touch on the whole suicide part but that’s actually really complicated even though we didn’t talk about it, right?  So you I don’t know if there’s any evidence but sometimes it is hard to judge how suicidal someone is.  And yeah I think definitely having clinical experience would allow you to use this more safely, yeah.” FP7  “In terms if you’re gonna roll it out online maybe even do like a video or like similar to that that first video in terms of how to click on things, but even just doing like a case scenario video.  I think would be helpful.” NP6 |
|  | Skills development: *The gradual acquisition or advancement through progressive stages of an ability or proficiency acquired through training and practice* |  |  |
|  | Competence: *One’s repertoire of skills, and ability especially as it is applied to a task or set of tasks* |  |  |
|  | Ability: *Competence or capacity to perform a physical or mental act. Ability may be either unlearned or acquired by education and practice* |  |  |
|  | Interpersonal skills: *An aptitude enabling a person to carry on effective relationships with others, such as an ability to cooperate, to assume appropriate social responsibilities or to exhibit adequate flexibility* |  |  |
|  | Practice: *Repetition of an act, behaviour, or series of activities, often to improve performance or acquire a skill* |  |  |
|  | Skills assessment: *A judgement of the quality, worth, importance, level, or value of an ability or proficiency acquired through training and practice* |  |  |
| **3. Social/Professional Role and ID**  *A coherent set of behaviours and displayed personal qualities of an individual in a social or work setting*  *How does who they are as a HCP influence whether they do something or not? (AP/JP)* | Professional identity: *The characteristics by which an individual is recognised relating to, connected with or befitting a particular profession* | Consider coding to this domain comments on:  Who uses or could use the algorithm  Specifically what each different health care provider, or colleague in the health care system, does as it relates to (or would relate to) using the algorithm  Consider also coding what participants usually do when using the algorithm (i.e. statements coded at Nature of Behaviour)  Any changes to the roles and responsibilities of any team members that use of the algorithm would require  More specific than social influence. A description of what someone else is doing (e.g., “Physicians diagnose” “Nurses discuss”)  Inappropriate coding to this domain:  Discussion of the need for other’s activity (e.g., nurse describing how they would need a physician to do something), code at ‘Social Influences’ instead | “I: Do you see like nurses, nurse practitioners using it?  R: Oh great yes they can sure absolutely yeah.” FP1  “R: Yeah it’ll give us more standardized so we have like we work in a big team, right. So there are 12 primary physicians and we’re, the nurse practitioners are seeing their patients. So if we have a more standardized approach it wouldn’t matter whether they’re seeing their primary provider or the nurse practitioner. People are gonna be treated in the same way.” NP4  “I: Do you think using the algorithm would require any changes to roles and responsibilities here and those could be your own or other team members?  R: You mean, no I mean nurse practitioners and doctors do the same thing. I don’t think we would change anything.” NP5 |
|  | Professional role: *The behaviour considered appropriate for a particular kind of work or social position* |  |  |
|  | Social identity: *The set of behavioural or personal characteristics by which an individual is recognizable [and portrays] as a member of a social group* |  |  |
|  | Identity: *An individual’s sense of self defined by a) a set of physical and psychological characteristics that is not wholly shared with any other person and b) a range of social and interpersonal affiliations (e.g., ethnicity) and social roles.* |  |  |
|  | Professional boundaries: *The bounds or limits relating to, or connected with a particular profession or calling* |  |  |
|  | Professional confidence: *an individual’s belief in his or her repertoire of skills and ability especially as it is applied to a task or set of tasks.* |  |  |
|  | Group identity: *the set of behavioural or personal characteristics by which an individual is recognizable [and portrays] as a member of a group* |  |  |
|  | Leadership: *The processes involved in leading others, including organising, directing, coordinating and motivating their efforts toward achievement of certain group or organization goals* |  |  |
|  | Organizational commitment: *An employee’s dedication to an organisation and wish to remain part of it. Organisational commitment is often described as having both an emotional or moral element and a more prudent element* |  |  |
| **4. Beliefs About Capabilities**  *Acceptance of the truth, reality, or validity about an ability, talent or facility that a person can put to constructive use*  *Do they think they can do what they should do and how does that influence whether they do it or not? (AP/JP)* | Self-confidence: *Self-assurance or trust in one’s own abilities, capabilities and judgement* | Consider coding to this domain comments on:  How easy or difficult it is/will be to use the algorithm  Confidence in ability to use the algorithm  Inappropriate coding to this domain:  Discussion of specific things that make the algorithm easy/difficult to use (this could be relevant to various domains depending on what is said) | “R: Well yes definitely the stress of being embarrassed about not being able to navigate like even just like figuring out how to email that. Okay well oh yeah I had to go back to that, you know, I could see being embarrassed with not being able to use it easily.” FP7  “How easy or difficult do you think it would be for you to use the algorithm in your practice?  R: Well fairly easy I think for sure, yeah” FP9 |
|  | Perceived competence: *An individual’s belief in her or her ability to learn and execute skills* |  |  |
|  | Self-efficacy: *An individual’s capacity to act effectively to bring about desired results, as perceived by the individual* |  |  |
|  | Perceived behavioural control: *an individual’s perception of the ease or difficulty of performing the behaviour of interest* |  |  |
|  | Beliefs: *The thing believed; the proposition or set of propositions held true* |  |  |
|  | Self-esteem: *The degree to which the qualities and characteristics contained in one’s self-concept are perceived to be positive* |  |  |
|  | Empowerment: *The promotion of the skills, knowledge and confidence necessary to take great control of one’s life as in certain educational or social schemes; the delegation of increase decision-making powers to individuals or groups in a society or organization* |  |  |
|  | Professional confidence: *An individual’s beliefs in his or her repertoire of skills, and ability, especially as it is applied to a task or set of tasks.* |  |  |
| **5. Optimism**  *The confidence that things will happen for the best or that desired goals will be attained*  *How does whether they are optimistic/ pessimistic influence what they do? (AP/JP)* | Optimism: *The attitude that outcomes will be positive and that people’s wishes or aims will be ultimately fulfilled* | Consider coding to this domain comments on:  Level of optimism regarding extent to which using the algorithm will help with depression care | “I think I’m pretty optimistic that it’ll be helpful.” NP4 |
|  | Pessimism: *The attitude that things will go wrong and that people’s wishes or aims are unlikely to be fulfilled* |  |  |
|  | Unrealistic optimism: *the inert tendency for humans to over-rate their own abilities and chances of positive outcomes compared to those of other people* |  |  |
|  | Identity |  |  |
| **6. Beliefs about Consequences**  *Acceptance of the truth, reality or validity about outcomes of a behaviour in a given situation*  *What are the good and bad things that can happen from what they do and how does that influence whether they’ll do it in the future? (AP/JP)* | Beliefs: *The thing believed; the proposition or set of propositions held true* | Consider coding to this domain comments on:  Positive and negative outcomes of using the algorithm (for self, patients, colleagues, and/or primary care setting)  How using the algorithm would or could be beneficial or not for the provision of depression care  The good or bad things that could potentially results from using the algorithm  Impact on workload:  How workload would or could be impacted by use of the algorithm (for self and others)  Consider also coding to reinforcement if includes reflection on previous experiences (must refer to those previous experiences impacting future consequences to be coded to beliefs about consequences) | “What do you think are would be the benefits or positive consequences that would come from using the algorithm do you think?  R: Well the patient info. Frankly I need a website that gives me the resources, right?  What do I where do I send this patient?” FP1  “R: I think it would increase my workload that’s the thing that worries me about that.  But if it improved my service to the patients then, you know, it would be worth it.  I: Mm hmm. And is the increase in workload related to just learning the detail that’s in it?  R: Learning how to use it, yeah.  I: Yeah mm hmm which might be a short-term problem but a long-term?  R: Gain yeah exactly.” FP7  “R: But no I don’t think that there’s harms like I don’t think I’m going to lose my ability to, you know, think for myself or not factor in all the contextual issues of their depression” NP2 |
|  | Outcome expectancies: *Cognitive, emotional, behavioural, and affective outcomes that are assumed to be associated with future or intended behaviour. These assumed outcomes can either promote or inhibit future behaviours.* |  |  |
|  | Characteristics of outcome expectancies: *Characteristics of the cognitive, emotional and behavioural outcomes that individuals believe are associated with future or intended behaviours and that are believed to either promote or inhibit these behaviours. These include whether they are sanctions/rewards, proximal/distal, valued/not valued, probable/improbable. Salient/not salient, perceived risks or threat*s. |  |  |
|  | Anticipated regret: *A sense of the potential negative consequences of a decision that influences the choice made: for example an individual may decide not to make an investment because of the feelings associated with*  *an imagined loss* |  |  |
|  | Consequents: *An outcome behaviour in a given situation* |  |  |
| **7. Reinforcement**  *Increasing the probability of a response by arranging a dependent relationship, or contingency, between the response and a given stimulus*  *How have their experiences (good and bad) of doing it in the past influence whether or not they do it? (AP/JP)* | Rewards (proximal/distal, valued/ not valued, probable/improbable): *Return or recompense made to, or received by a person contingent on some performance.* | Consider coding to this domain:  Discussion of reinforcement/rewarding experiences that would encourage use of the algorithm (may be based on past experience but likely hypothetical)  Discussion of sanctions/negative experiences/ deterrents/ discouragements that would discourage use of the algorithm (may be based on past experience but likely hypothetical)  Discussion of previous positive/negative experiences with other algorithms (if explicitly related to use of this algorithm)  Note: consider also coding to beliefs about consequences if includes projection to future consequences (but must refer to previous experiences/hypothetical rewarding or punishing experiences to be coded to reinforcement) | “I think the probably the most useful resource in here would be a the initial handouts of describing what depression is to a patient if the patient didn’t know what was wrong with them.  So reading through that and being aware of that and deciding if that was something that was reassuring and helpful for patients I could see if I had if I used that once or twice and the patients say, you know, thank you for giving me that information that really helped me to understand what was going on. Then that would make me want to use it more and the same with the patient education area, you know, if I found a page or a product that I got feedback from a patient that they found it helpful then I would be okay I should use that again, yeah.” FP7  “Are there any do you think sort of incentives or kind of rewarding type experiences that would encourage you to use the algorithm? It’s kind of a tricky one so different from sort of positive things that could come from it, you know some things that would maybe reinforce you to use it.  R: Yeah that’s a hard one. I don’t really think so, no.” R2 |
|  | Incentives: *An external stimulus, such as condition or object, that enhances or serves as a motive for behaviour* |  |  |
|  | Punishment: *The process in which the relationship between as response and some stimulus or circumstance results in the response becoming less probable; a painful, unwanted or undesired event or circumstance imposed as a penalty on a wrongdoer* |  |  |
|  | Consequents: *An outcome of behaviour in a given situation* |  |  |
|  | Reinforcement: *A process in which the frequency of a response is increased by a dependent relationship or contingency with a stimulus* |  |  |
|  | Contingencies: *A conditional probabilistic relation between two events. Contingencies may be arranged via dependencies or they may emerge by accident* |  |  |
|  | Sanctions: *A punishment or other coercive measure, usually administered by a recognized authority, that is used to penalise and deter inappropriate or unauthorized actions.* |  |  |
| **8. Intention**  *A conscious decision to perform a behaviour or a resolve to act in a certain way*  *How does how inclined they are to do something influence whether they will do it? (AP/JP)* | Stability of intentions: *ability of one’s resolve to remain in spite of disturbing influences* | Consider coding to this domain comments on:  How motivated they are to/how much they want to use the algorithm in general (consider also coding to ‘nature of behaviour’ if includes discussion of specific situations in which algorithm is/would be used) (consider also coding to ‘memory, attention and decision processes’ if decision-making regarding using the algorithm is discussed)  Participant’s descriptions of how inclined they are to use the algorithm in general (consider also coding to ‘nature of behaviour’ if includes discussion of specific situations in which algorithm is/would be used) (consider also coding to ‘memory, attention and decision processes’ if decision-making regarding using the algorithm is discussed)  **Note:** Indicator of intention must be explicit and not inferred  Inappropriate coding to this domain: this is different from how effective they think using the algorithm will be, and different from whether they think using the algorithm will result in positive outcomes (‘Beliefs About Consequences’) and different from how much of a priority using the algorithm is for them (‘Goals’).  Be careful not to code the reasons for the intention (focus on statements that directly reflect their intention and motivation) | “I: And to what extent would you say you want to use the algorithm going forward?  R: Yeah I’m not sure on a scale of 1-10 or [laugh] but however you want it, but I would say like a 9 on 10, but I would definitely be optimistic to use it and willing to use it.” FP9  ‘R: Probably I wouldn’t go to algorithm to use if for example, the patient who come is one problem and in between mentions something about depression probably I wouldn’t go to the algorithm because it’s time consuming. But if the patient comes in visit mainly because as a depression, yeah I definitely would use this algorithm.’ NP7 |
|  | Stages of Change model: *A model that proposes that behaviour change is accomplished through five specific stages* |  |  |
|  | Transtheoretical model and stages of change: *a five-stage theory to explain changes in people’s health behaviour. It suggests that change takes time, that different interventions are effective at different stages, and that there are multiple outcomes occurring across the stages* |  |  |
| **9. Goals**  *Mental representations of outcomes or end states that an individual wants to achieve*  *How important is what they do and does that influence whether or not they do it? What standards are they trying to reach, how does that influence whether or not they do it? (AP/JP)* | Goals (distal/proximal): *Desired state of affairs of a person or system, these may be closer (proximal) or further away (distal)* | Consider coding to this domain:  Descriptions of whether or not using the algorithm is a priority  Descriptions of extent to which using the algorithm is a priority compared to other priorities during the consultation  Consider coding awareness of guidelines/local policies, or lack of guidelines/local policies for screening/diagnosing/managing depression (also code at knowledge)  Consider coding description of complementarity/conflict between policy/guidelines and the algorithm (also code at knowledge)  Consider coding description of how algorithm adds to/does not add to current policy/guidelines (also code at knowledge)  Descriptions of how using the algorithm is (or is not) in conflict with guidelines/local policies currently used (also code at knowledge) or in conflict with other aspects of the care they provide (goal conflict)  Practice standards (also code at knowledge) | “R: I think it would be a pretty high priority because it helps guide. So once you’re used to it it’s gonna be pretty quick and could actually save you time rather than having like having everything on one page or one website not going back and forth.” NP4  “And for general anxiety/depression disorders I follow the Up-to-Date guidelines and we all have Up-to-Date here so those are updated regularly.” NP5  “R: Well, you know, it’s the trouble with these algorithms is that they’re very cumbersome, right?  Like we don’t use algorithms. As I said it’s great for teaching residents and stuff like I have to deal with another 5 issues of this lady. So for me to pull this out as I said the only time I open this is when I’m changing meds or I’m stuck with a non-responder, right?” FP1 |
|  | Goal priority: *Order of importance or urgency of end state toward which one is striving* |  |  |
|  | Goal/target setting: *A process that establishes specific time based behavioural targets that are measureable, achievable and realistic* |  |  |
|  | Goals (autonomous/controlled): *The end state toward which one is striving: the purpose of an activity or endeavour. It can be identified by observing that a person ceases or changes their behaviour upon attaining this state; proficiency in a task to be achieved within a set period of time.* |  |  |
|  | Action planning: *The action or process of forming a plan regarding a thing to be done or a deed* |  |  |
|  | Implementation intention: *The plan that one creates in advance of when, where an how one will enact a behaviour* |  |  |
| **10. Memory, Attention and Decision Processes**  *The ability to retain information, focus selectively on aspects of the environment and choose between two or more alternatives*  *How does their forgetfulness or remembering to do it influence whether or not they actually do it? How does their ability to focus on the behaviour influence whether or not they do it? How do the decision they make about the behaviour influence whether they do it or not? (AP/JP)* | Memory: *The ability to retain information or a representation of a past experience, based on the mental processes of learning or encoding retention across some interval of time, and retrieval or reactivation of the memory; specific information of a specific task* | Consider coding to this domain:  When/why would it be easy to forget to use the algorithm.  Due the hypothetical nature of the interviews, please also code participant’s descriptions of when they think they would forget as well as reasons why they don’t think they would forget at this domain  Descriptions of decision/thought processes regarding using the algorithm  Discussion of specific situations in which algorithm is/would be used (if decision-making regarding using the algorithm is explicitly discussed) (also code to nature of behaviour if relevant)  Question from guide ‘What could help you to integrate the use of the algorithm into your routine or habits?’ responses likely more relevant to other domains | “and then forgetting wise it might be for the ones you know well. The patients you know really well and  I: Mm hmm you’re sort of in that routine already.  R: In that routine which is sometimes the routine is a bad thing sometimes you miss things, right, so it’s good to have this in that situation but definitely forgetting to use it in that situation, yeah.” FP9  “R: Have a patient, known her for 20 years, she’s had a couple episodes of depression over those 20 years that have responded to a particular type of therapy and she’s gone on to do well for many years. And then she relapses when there’s some big crisis in her life.  I can probably not argue with success I would probably go back to what we had done before if it had worked really well.  So that would be one where I probably wouldn’t think very much of using the algorithm.” FP3 |
|  | Attention: *A state of awareness in which the senses are focussed selectively on aspects of the environment and the central nervous system is in a state of readiness to respond to stimuli* |  |  |
|  | Attention control: *The extent to which a person can concentrate on relevant cues and ignore all irrelevant cues in a given situation* |  |  |
|  | Decision making: *The cognitive process of choosing between two or more alternatives, ranging from the relatively clear-cut to the complex* |  |  |
|  | Cognitive overload/tiredness: *The situation in which the demands placed on a person by mental work are greater than a person’s mental abilities* |  |  |
| **11. Environmental Context and Resources**  *Any circumstance of a person’s situation or environment that discourages or encourages the development of skills and abilities, independence, social competence, and adaptive behaviour*  *What are the things in their environment that influence what they do and how do they influence?(not just physical stuff, but access to other professionals) (AP/JP)* | Environmental stressors*: External factors in the environment that cause stress* | Consider coding to this domain:  Resources needed to use algorithm, and extent or availability of these (also code descriptions of use of resources at ‘Behaviour Regulation’)  Discussion of aspects of environment which might influence likelihood of using algorithm  Descriptions of software/EMR used  Context of the practice itself that would/does influence whether algorithm used.  Descriptions of how more time will be required for use of the algorithm  Descriptions of concerns regarding how the information in the algorithm will be kept up-to-date in accordance with evidence-based practice (not knowledge as issue is not their knowledge per se; not social influences unless specific others are mentioned) | “to what extent would you say it would be something that you would want to implement in your practice?  R: I would say to the, it goes back to the same comment that I made, it would be to the extent that the special services are available to access.  Because what we find is while I see this as great conceptually our in practice experience is not this and so then we are left scrambling with the chronic, with the medium, managing as best we can until they’re in crisis and we send them to the hospital.  That’s, that’s our experience. So and those would be huge roadblocks for us to implement it as it is.” A1  “if I don’t have access to my computer. I do home visits so sometimes I don’t my the Wi-Fi doesn’t work so I would say 50% of the time I don’t have a computer. So then I wouldn’t be able to access it. I would probably use it not to initiate treatment but to verify treatment after.  But still would be able to use it afterwards.” NP5  “If this algorithm can be integrated inside of the PSS system, our PSS system, it’s probably would make it more useful than if I need to go to the independent site and go to the algorithm from outside yeah.” NP7  “I: And so just thinking about everything we’ve talked about today what are the most important factors would you say that influence you using the algorithm? What are the sort of main drivers that encourage you to use it?  R: So usability of the interface which is mostly there. Quality of the resources which is there. Let’s see mm and then I suppose the other element that I brought up earlier is currently it’s evidence-based [laugh], provided it continues to remain so, it’s a good resource. Because again this is replacing probably Up-to-Date as a resource for most physicians and that’s by nature is, is kept up-to-date.” R1 |
|  | Resources/material resources: *Commodities and human resources used in enacting a behaviour* |  |  |
|  | Organizational culture/climate: *A distinctive pattern of thought and behaviour shared by members of the same organization and reflected in their language, values, attitudes, beliefs and customs* |  |  |
|  | Salient events/critical incidents: *Occurrences that one judges to be distinctive, prominent or otherwise significant* |  |  |
|  | Person x environment interaction: *Interplay between the individual and their surroundings* |  |  |
|  | Barriers and facilitators: *In psychological contexts, barriers/facilitators are mental, emotional or behavioural limitations/strengths in individuals or groups* |  |  |
| **12.Social Influences**  *Those interpersonal processes that can cause individuals to change their thoughts, feelings, or behaviours*  *What do others think of what they do? Who are they and how does that influence what they do? (AP/JP)* | Social pressure: *the exertion of influence on a person or group by another person or group* | Consider coding to this domain:  Discussion about how others influence whether or not the algorithm is/would be used. Includes patient influence, and influence of other healthcare professionals.  Discussion of the need for other’s activity (e.g., nurse describing how they would need a physician to do something)  Descriptions of how nurses follow doctor’s orders  Descriptions of practice-wide pattern of thought related to use of the algorithm (considered social norms)  Inappropriate coding to this domain:  Specific descriptions of the roles of others i.e. what someone else is doing “Physicians diagnose” “Nurses discuss” should be coded at ‘Social Professional Role and Identity.’ | “R: Well I think if my colleagues were using it I’d be more likely to use it yeah.” FP7  “Is there any other people who might influence your likelihood of using the algorithm be it like your colleagues or even your patients maybe?  R: So absolutely because they’re not rostered to me so if the physician’s preference is not to use that that’s their patient so that would certainly be something that I would have to consider.” NP6  “And is there any people who would influence whether you use the algorithm so that could be colleagues, patients and others?  R: Hmm probably not. Only if I my personal level of comfort to use the algorithm what is it can affect my willing to use not to use, yeah.” NP7 |
|  | Social norms: *Socially determined consensual standards that indicate a) what behaviours are considered typical in a given context and b) what behaviours are considered proper in the context* |  |  |
|  | Group conformity: *The act of consciously maintaining a certain degree of similarity to those in your general social circles* |  |  |
|  | Social comparisons: *The process by which people evaluate their attitudes, abilities or performance relative to others* |  |  |
|  | Group norms: *Any behaviour, belief, attitude or emotional reaction held to be correct or acceptable by a given group in society* |  |  |
|  | Social support: *The apperception or provision of assistance or comfort to others, typically in order to help them cope with a variety of biological, psychological and social stressors. Support may arise from any interpersonal relationship in an individual’s social network, involving friends, neighbours, religious institutions, colleagues, caregivers of support groups* |  |  |
|  | Power: *The capacity to influence others, even when they try to resist this influence* |  |  |
|  | Intergroup conflict: *Disagreement or confrontation between two or more groups and their members. This may involve physical violence, interpersonal discord, or psychological tension.* |  |  |
|  | Alienation: *Estrangement from one's social group; a deep seated sense of dissatisfaction with one's personal experiences that can be a*  *source of lack of trust in one's social or physical environment or*  *in oneself; the experience of separation between thoughts and*  *feelings* |  |  |
|  | Group identity: *the set of behavioural or personal characteristics by which an individual is recognizable [and portrays] as a member of a group* |  |  |
|  | Modelling: *In developmental psychology the process in which one or more individuals or other entities serve as examples (models) that a child will copy* |  |  |
| **13. Emotion**  *A complex reaction pattern, involving experiential, behavioural and physiological elements, by which the individual attempts to deal with a personally significant matter or event*  *How do they feel about what they do and do those feelings influence what they do? (AP/JP)* | Fear: *An intense emotion aroused by the detection of imminent threat, involving an immediate alarm reaction that mobilizes the organism by triggering a set of physiological changes* | Consider coding to this domain comments on:  Emotions experienced (or anticipated) when using the algorithm (can be positive or negative)  When participant would be worried/concerned about using the algorithm  Inappropriate coding to this domain:  Descriptions of patients’ emotions regarding use of the algorithm (code at ‘Social Influences’ instead). | How would you say you feel about using the algorithm so what do any kind of emotions come to mind when you think about using it be that sort of positive emotions or is there any like stress?  R: No I’m actually quite excited.” NP5  “R: Well yes definitely the stress of being embarrassed about not being able to navigate like even just like figuring out how to email that. Okay well oh yeah I had to go back to that, you know, I could see being embarrassed with not being able to use it easily.” FP7 |
|  | Anxiety: *A mood state characterized by apprehension and somatic symptoms of tension in which an individual anticipates*  *impending danger, catastrophe or misfortune.* |  |  |
|  | Affect: *An experience or feeling of emotion, ranging from suffering to*  *elation, from the simplest to the most complex sensations of*  *feelings, and from the most normal to the most pathological*  *emotional reactions.* |  |  |
|  | Stress: *A state of physiological or psychological response to internal or external stressors* |  |  |
|  | Depression: *A mental state that presents with depressed mood, loss of interest or pleasure, feelings of guilt or low self-worth, disturbed sleep or appetite, low energy, and poor concentration* |  |  |
|  | Positive/negative affect: *the internal feeling/state that occurs when a goal has/has not been attained. A source of threat has/has not been avoided, or the individual is/is not satisfied with the present state of affairs* |  |  |
|  | Burn-out: *Physical, emotional or mental exhaustion, especially in one’s job or career, accompanied by decreased motivation, lowered performance and negative attitudes towards oneself and others* |  |  |
| **14. Behavioural Regulation**  *Anything aimed at managing or changing objectively measured actions*  *What do they think would help them/what strategies have helped them do what you should do? What strategies are already in place to help them do what they should do? (AP/JP)* | Self-monitoring: *A method used in behavioural management in which individuals keep a record of their behaviour, especially in connection with efforts to changes or regulate the self; a personality trait reflecting an ability to modify one’s behaviour in response to a situation* | Consider coding to this domain:  Self-regulatory strategies that would influence the use of the algorithm. Focus on self-regulatory strategies only (not all strategies) (i.e. intention formation, goal setting, feedback on behaviour, self-monitoring of behaviour, review of behavioural goals)  Action plans relating to use of the algorithm  Coping plans/problem-solving strategies to mitigate potential problems with using the algorithm  Description of use of resources needed to use algorithm (also code at environmental context and resources)  Inappropriate coding to this domain: strategies suggested by the participant as being useful to implement the use of the algorithm more broadly. This would be ‘Strategies Suggested for Implementation.’ | “R: Well I, I do have it integrated into my personal bookmarks.” FP10  “is there anything you think could help you integrate the use of the algorithm into your routines and habits?  R: I think just having the icon on your desktop.  I: Mm hmm yeah good.  R: So it’s really easily accessible.” NP1 |
|  | Breaking habit: *to discontinue a behaviour or sequence of behaviours that is automatically activated by relevant situational cues* |  |  |
|  | Action planning: *The action or process of forming a plan regarding a thing to be done or a deed.* |  |  |
| **15. Nature of Behaviour**  *What do you do and is that different from what you should do? (AP/JP)* |  | Consider coding to this domain:  What participants usually do when using the algorithm (consider also coding to Social Professional Role and Identity)  Discussion of specific situations in which algorithm is/would be used/situations in which participants could see themselves using the algorithm (consider also coding to ‘intention’ if include discussion of how inclined/motivated they are to use the algorithm) (consider also coding to ‘memory, attention and decision processes’ if decision-making regarding using the algorithm is discussed)  Descriptions of how/where/when algorithm is/would be used (consider also coding to ‘intention’ if include discussion of how inclined/motivated they are to use the algorithm) (consider also coding to ‘memory, attention and decision processes’ if decision-making regarding using the algorithm is discussed)  Descriptions of how participants are/are not currently using the algorithm.  Descriptions of how often the algorithm is used in usual practice  Also code to ‘Familiarity with or use of the ODA before participating’ if relevant (see descriptive information coding guide above) | “R: Most of the time I use it is when I’m going to second line treatment or if I’m switching.  I: Okay so second line is then you’ve tried something that hasn’t quite worked?  R: Something and then they come back and it’s not doing well and so now my question is should I go up on this med? Should I completely switch or should I add something?  And that’s where I sometimes do.  I: So it’s mostly around the medication then?  R: Mostly around medication I must say.” FP1  “And if I was using it in front of a patient and not doing a good job and it maybe would make them have less confidence [laugh] in, in me as a provider.  So I might not use it in front of the patients.” FP7  “R: I would use it for everyone, for anyone that’s like I do for the PHQ9 and other the GAD and the other tools that I use I would probably get this as another tool and use it regularly.” NP5 |
