# Supplementary material for: Combining theory and usability testing to inform optimization and implementation of an online primary care depression management tool
Source: BMC Med Inform Decis Mak. 2025 Jan 15;25:25. doi: 10.1186/s12911-024-02733-7 (PMC11734350; doi:10.1186/s12911-024-02733-7)
Supplement: Supplementary file 4 — Supplementary Material 4. [file 12911_2024_2733_MOESM4_ESM.docx]

**Combining theory and usability testing to inform optimization and implementation of an online primary care depression management tool**

**Additional File 4: Usability categories and TDF domains not included in reported themes**

**Table s1. Usability testing – usability categories not included in reported themes**

| **Category** | **Rationale for exclusion** |
| --- | --- |
| Search | No statements coded here |
| Graphics | No statements coded here |
| Colour | No statements were grouped as having shared meaning around a central concept |
| Hardware or software | One common issue identified: slow internet connection impacts usability: reported in section on barriers to and enablers of algorithm use in day-to-day practice |
| Consistency of operations | No statements were grouped as having shared meaning around a central concept |

**Table s2. Barriers to and enablers of algorithm use in day-to-day practice – TDF domains not included in reported themes**

| **Domain** | **Rationale for exclusion** |
| --- | --- |
| Skills | Participants did not identify any skills deficits or additional skills required to use the algorithm other than basic IT skills and relevant clinical skills which they and other intended users would already possess |
| Beliefs about capabilities | Most participants did not identify any issues with their capability to use the algorithm; the few issues identified would be mitigated by tackling the larger barriers (e.g. getting to know the algorithm more would make it easier to use) |
| Optimism | Most participants were generally optimistic about the algorithm, and did not highlight significant impacts of this on their use of the algorithm |
| Reinforcement | Few statements were grouped as having shared meaning around a central concept; possible reinforcements identified but these were conditional upon gaining more experience with using the algorithm (since most participants had limited or no experience) |
| Emotion | Few statements referred to the same emotions; the few negative emotional issues highlighted were largely hypothetical (since most participants had limited or no experience using the algorithm) and would be mitigated by tackling the larger barriers (e.g. getting to know the algorithm more would reduce discomfort with using it) |

Notes: TDF = Theoretical Domains Framework
